# Sprint‐interval training with post‐exercise blood flow restriction increases mitochondrial content and respiration

**DOI:** 10.1113/JP290955

**Published:** 2026-06-03

**Authors:** Donald L. Peden, Carolin Stangier, Emma A. Mitchell, Stephen J. Bailey, Richard A. Ferguson

**Affiliations:** ^1^ School of Sport, Exercise and Health Sciences Loughborough University Loughborough UK; ^2^ David Greenfield Human Physiology Unit, School of Life Sciences University of Nottingham Nottingham UK

**Keywords:** biogenesis, exercise performance, high intensity, HIIT, local hypoxia, SIT

## Abstract

**Abstract:**

Sprint interval training (SIT) combined with post‐exercise blood flow restriction (BFR) can augment adaptive signalling responses in skeletal muscle. However, mitochondrial adaptations to SIT with BFR are not well‐understood. This study examined the effects of a 6 week SIT program with or without post‐exercise BFR on skeletal muscle mitochondrial content and respiratory function, alongside physiological performance markers. Physically active males (*n = *20; 25.3 ± 5.9 years; ${\dot{V}}_{O_{2}\mathrm{peak}}$, 52.5 ± 4.6 mL·min^−1^·kg^−1^) completed a SIT intervention (repeated 30 s sprints interspersed with 4.5 min of rest) with (BFR; *n = *12) or without (CON; *n = *8) post‐exercise BFR. Baseline and post‐training ${\dot{V}}_{O_{2}\mathrm{peak}}$ and lactate thresholds were measured and muscle biopsies obtained for determination of citrate synthase (CS) activity and mitochondrial respiration [O_2_ flux during leak (_L_), ADP‐stimulated oxidative phosphorylation (_P_) and uncoupled maximal electron transfer (_E_) states through mitochondrial complexes I–IV (CI–IV)]. There were time × condition interactions for CS activity (*P = *0.011) and CS activity‐corrected CII_E_ (*P = *0.047) and CIV_E_ (*P = *0.010), which increased following BFR (12.1%, *P = *0.040; 74.3%, *P = *0.030; 64.4%, *P = *0.002, respectively) but not in CON (−4.6%, *P = *0.053; 9.9% *P = *0.460; −7.4%, *P = *0.664, respectively). There were no between‐group differences in the changes in ${\dot{V}}_{O_{2}\mathrm{peak}}$ or any other performance markers (*P *≥ 0.176). The addition of BFR to a 6 week SIT program increased mitochondrial content and uncoupled respiration in physically active males, which may have implications for improving skeletal muscle oxidative metabolism.

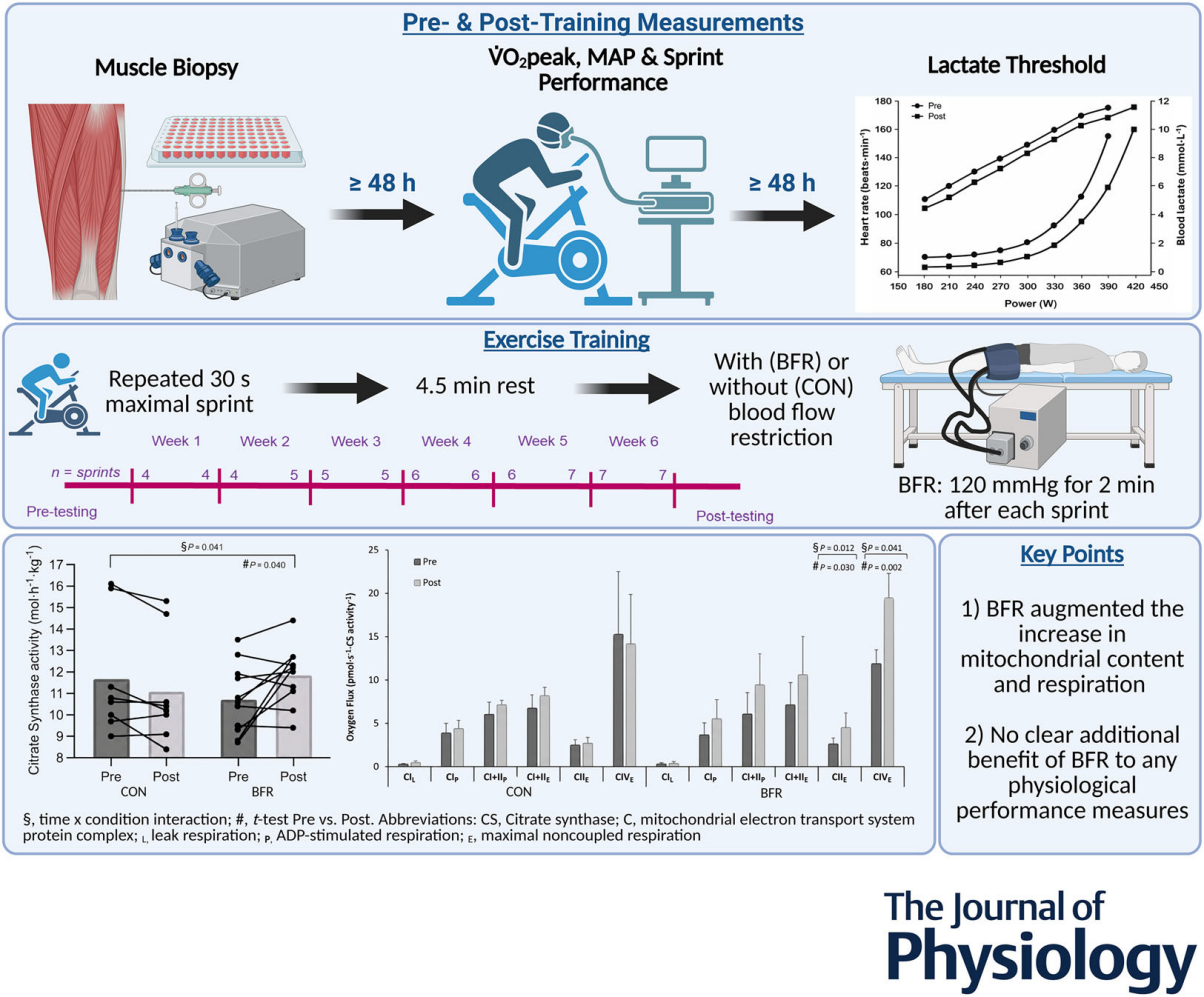

**Key points:**

- Sprint interval training (SIT) with post‐exercise BFR (SIT+BFR) augments the exercise stimulus without limiting intensity and is shown to improve maximal oxygen uptake in athletes compared to SIT alone. However, the mechanisms underpinning this response remain unclear.
- This study is the first to investigate the effects of SIT+BFR on citrate synthase (CS) activity and mitochondrial respiratory parameters.
- We demonstrate that a 6 week SIT+BFR intervention in physically active males, increased CS activity and uncoupled mitochondrial respiration compared to SIT alone. However, improvements in performance determinants in response to SIT did not differ between groups.
- These findings provide novel insight into the mitochondrial bioenergetic potential of BFR, when combined with SIT, and can inform recommendations for exercise training interventions.

## Graphical abstract

**Abstract figure legend** The mechanisms underpinning improved maximal oxygen uptake following sprint interval training (SIT) with post‐exercise blood flow restriction (BFR), compared to SIT alone, remain unclear. This study examined the effects of 6 week SIT with (BFR; *n = *12) or without (CON; *n = 8*) post‐exercise BFR on ${\dot{V}}_{O_{2}\mathrm{peak}}$, lactate thresholds, citrate synthase (CS) activity and mitochondrial respiration in physically active males. There were time × condition interactions for CS activity (*P = *0.011) and CS activity‐corrected CII_E_ (*P = *0.047) and CIV_E_ (*P = *0.010), which increased following BFR (12.1%, *P = *0.040; 74.3%, *P = *0.030; 64.4%, *P = *0.002, respectively) but not in CON (−4.6%, *P = *0.053; 9.9% *P = *0.460; –7.4%, *P = *0.664, respectively). There were no between‐group differences in any performance marker changes (*P *≥ 0.176). The addition of BFR to a 6 week SIT program increased mitochondrial content and uncoupled respiration.

## Introduction

The key physiological determinants of endurance performance, such as ${\dot{V}}_{O_{2}\max}$, lactate threshold (LT) and critical power, are closely associated with mitochondrial capacity (Granata et al., 2018; Hoppeler et al., 1973; Peden et al, 2025; Van Der Zwaard et al., 2016). It is well‐documented that adaptations in both mitochondrial content and respiratory function can be achieved with exercise training (Bishop et al., 2025; Holloszy, 1967). Training‐induced adaptations are governed by the physiological and metabolic stressors associated with exercise, such as increases in AMP/ATP ratio and cytosolic calcium concentration, as well as altered oxidative stress and redox state (Egan & Zierath, 2013). These stressors initiate molecular signalling cascades that increase the abundance and/or function of proteins, which contributes to improved mitochondrial capacity after exercise training (Egan & Zierath, 2013; Hood, 2009; Ljubicic et al., 2010).

Training‐induced adaptations of the mitochondria appear to depend on characteristics of the exercise stimulus (Bishop et al., 2025; Laursen & Jenkins, 2002; MacInnis & Gibala, 2017), with considerable debate regarding primacy of exercise intensity and training volume to improve mitochondrial function and content (Bishop et al, 2019; MacInnis et al., 2019). A recent systematic review and meta‑regression analysis suggests both larger training volumes and higher training intensities are associated with increases in mitochondrial content (Mølmen et al., 2025). However, manipulations of training intensity and volume differentially influence mitochondrial respiration (Bishop et al., 2025; Granata et al., 2016) with intensity appearing to be more important for training‐induced improvements in mitochondria‐specific respiration. This is potentially the result of increased homeostatic perturbations arising from high‐intensity exercise driving mitochondrial signalling cascades, mitophagy and consequential mitochondrial turnover (Botella et al., 2025; Granata et al., 2016; Li et al., 2025). It is also well‐established that mitochodrial adaptations are largely determined by initial fitness level (Mølmen et al., 2025) with blunted adaptive signalling responses to exercise observed in trained compared to untrained individuals (McConnell et al., 2020) or with a repeated exercise stimulus (Granata et al., 2020; Perry et al., 2010). In trained populations, exercise intensity is also essential to induce and maintain skeletal muscle adaptations and performance (Bangsbo et al., 2025; Laursen & Jenkins, 2002; Laursen et al., 2005). Therefore, interventions that can augment physiological stress and subsequent signalling responses to exercise, without limiting training volume or intensity, may overcome the blunting of adaptive responses in trained individuals.

Exercise combined with blood flow restriction (BFR) is one potential strategy to alter the physiological stressors associated with training adaptation. Research shows that exercising with BFR can increase skeletal muscle metabolic demand (Suga et al., 2009, 2012; Takarada et al., 2000) and oxidative stress (Christiansen et al., 2018; Corvino et al., 2017; Downs et al., 2014; Karabulut et al., 2011). BFR during low‐intensity running has been demonstrated to increase AMP‐acitvated protein kinase (AMPK) signalling and peroxisome proliferator‐activated receptor gamma coactivator 1‐alpha (PGC‐1α) mRNA expression (Christiansen et al., 2018). However, enhanced signalling responses from low‐intensity exercise with BFR are comparable to those induced by high‐intensity exercise alone (Biazon et al., 2019; Groennebaek et al., 2018). Moreover, Christiansen et al. (2020) found no changes in mitochondrial oxidative phosphrylation in response to low‐intensity training with BFR. Research shows that utilising BFR during the rest periods of sprint interval training (SIT) does not limit session exercise intensity or overall training volume (Mitchell et al., 2019; Taylor et al., 2016). However, evidence regarding upregulation of adaptive signalling responses and mitochondrial biogenesis, as well as the translation to performance adaptations, when combining SIT with BFR is contradictory (Ferguson et al., 2021). For example, hypoxia‐inducible factor‐1α mRNA expression increased by a greater extent following SIT with BFR compared to SIT alone; however, there were no differences in AMPK activation or PGC‐1α mRNA expression between conditions (Taylor et al., 2016). Moreover, there were no changes in citrate synthase (CS), cytochrome *c* oxidase (COX)II and COXIV protein content following 4 weeks of SIT with BFR (Mitchell et al., 2019). This was despite an increased ${\dot{V}}_{O_{2}\max}$ of ∼5%, that was not observed with SIT alone. The lack of training‐induced increase in mitochondrial content markers may reflect the fact that measurement of mitochondrial protein is a poor biomarker of overall mitochondrial content (Larsen et al., 2012). Furthermore, the potential effect of SIT with BFR on mitochondrial respiration is unknown.

The main aim of the present study was to investigate the effects of a 6 week cycling SIT intervention combined with post‐exercise BFR on markers of mitochondrial content and respiratory function. It was hypothesised that SIT with BFR would result in a greater increase in CS activity and mitochondrial respiration, expressed relative to tissue mass and CS activity, in healthy active individuals, compared to SIT alone. It was also hypothesised that SIT with BFR would augment training‐induced increases in cycling ${\dot{V}}_{O_{2}\mathrm{peak}}$ and LT parameters.

## Methods

### Participants

Twenty healthy males (age: 25 ± 6 years, height: 1.80 ± 0.59 m, body mass: 81.7 ± 10.4 kg) volunteered to participate in this study. All participants were completing at least three endurance‐based exercise sessions per week and had a minimum cycling experience of 1 year. All participants completed health screening questionnaires prior to participation to mitigate for contraindications to maximal exercise, BFR and muscle biopsy procedures. Participants did not have a history of neuromuscular, haematological, musculoskeletal abnormalities or allergy to lidocaine hydrochloride administration, and were not using pharmacological treatments during the study period. Participants were fully informed of the risks and discomforts associated with all experimental procedures before providing written, informed consent. All experimental procedures were approved by the Loughborough University Ethics Approvals Human Participants Sub‐Committee (R16‐P096 and R19‐P256) and conformed to the *Declaration of Helsinki*, except for registration in a database.

### Experimental design

Participants were assigned to one of two groups in an independent groups study design, completing either a 6 week SIT protocol combined with (BFR, *n* = 12) or without (CON, *n* = 8) post‐exercise blood flow restriction. Participants were allocated to intervention groups in a deterministic, pair‐matched fashion based upon initial ${\dot{V}}_{O_{2}\mathrm{peak}}$ and maximal aerobic power (MAP) determined during preliminary testing. Participants were initially familiarised to the testing and training procedures during a preliminary visit, which also served to determine whether the performance inclusion criteria were met (${\dot{V}}_{O_{2}\mathrm{peak}}$ ≥ 50 mL·min^−1^·kg^−1^). Subsequently, pre‐training outcome measures were assessed across three laboratory visits, each separated by at least 48 h. During the first visit, a resting muscle biopsy was obtained. On the second visit, participants completed a maximal sprint and an incremental exercise test to determine ${\dot{V}}_{O_{2}\mathrm{peak}}$ and MAP. On the third visit, participants completed a submaximal exercise test to determine LT parameters. Participants then underwent a 6 week supervised training programme. Approximately 3–5 days following the final training session, post‐training outcome measures were assessed in the same order and over a similar time‐period as pre‐training.

All pre‐ and post‐training exercise tests were performed on an electronically braked cycle ergometer (Lode Excalibur Sport, Lode BV, Groningen, The Netherlands). Handlebar height and reach, saddle height, and fore‐aft dimensions were self‐selected by the participant, recorded for each participant during the preliminary testing session and remained constant for all subsequent visits. Participants were instructed to maintain a normal diet, including any supplements, during the pre‐training testing and to replicate that diet during the post‐training measures. Participants were instructed to refrain from ingesting alcohol and caffeine during the 48 h preceding testing. Exercise trials were undertaken at approximately the same time each day (±2 h). Laboratory conditions during pre‐ and post‐training exercise measurements remained constant (19–21°C and 40–50% humidity).

### Pre‐ and post‐training outcome measures

#### Maximal sprint

Participants performed a 20 s maximal sprint on the cycle ergometer from a stationary start. Following a 10 min warm up at 75 W, the 20 s sprint was performed against a fixed manufacturer‐specific‐torque‐factor setting (0.7; Wingate Anaerobic Test, Lode B.V.) relative to the participant body mass (torque factor x body mass = braking torque (N·m^−1^). Peak power output (PPO), mean power output (MPO) and minimum power output (MIN) were determined at a sampling rate of 5 Hz, with the highest and lowest single values taken for PPO and MIN, respectively.

#### ${\dot{V}}_{O_{2}\mathrm{peak}}$ and MAP

Participants rested in a seated position for 45 min following the maximal sprint test and subsequently performed a ramp incremental test to exhaustion to determine ${\dot{V}}_{O_{2}\mathrm{peak}}$ and MAP. Participants began cycling, at a freely chosen constant pedal cadence at a starting power of 25 W for 3 min, after which power was increased by 25 W·min^−1^ (0.42 W·s^−1^) until volitional exhaustion or when the selected pedal cadence dropped by 10% for more than 5 s, despite strong verbal encouragement. Breath‐by‐breath pulmonary gas exchange was measured continuously throughout exercise (Vyntus CPX; Jaeger‐Carefusion, Höchberg, Germany) via a facemask (Hans‐Rulolph Oro‐nasal 7450 V2; Cranlea Human Performance Ltd, Birmingham, U.K.), mouthpiece and impeller turbine assembly (Jaeger Triple V; Jaeger‐Carefusion). Prior to each visit, the system was calibrated with gas of known concentration (16% O_2_ and 5% CO_2_), with the turbine volume transducer calibrated using a 3 L syringe (Hans Rudolph, Kansas City, KS, USA). ${\dot{V}}_{O_{2}\mathrm{peak}}$ was defined as the highest ${\dot{V}}_{O_{2}}$ sustained for 30 s, with MAP defined as the highest power output achieved during the final min of the incremental test.

#### LT

Participants performed a step incremental test, at a freely chosen pedal cadence, consisting of 4 min stages starting at ∼100 W, which was adjusted based on MAP achieved during the ramp test to exhaustion. Power output at each stage was increased by 25 W, which continued for a minimum of 8 stages until a heart rate ≥ 90% of the maximum heart rate achieved during the ${\dot{V}}_{O_{2}\mathrm{peak}}$ test. Capillary blood samples (20 µL) were obtained from the earlobe during the last 30 s of each stage and immediately placed in homogenising solution line (EKF Diagnostics, Cardiff, UK). Blood lactate concentration (B[La]) was determined (C‐line Biosen; EKF Diagnostics) within 1 h of sampling. A lactate‐power curve was produced for each participant with LT parameters defined as: power output corresponding to an initial increase of 1 mmol·L^−1^ B[La] (LT 1 mmol·L^−1^) (Thoden et al., 1991), a fixed 4 mmol·L^−1^ B[La] (FBLA_4_) (Kindermann et al., 1979) and Dmax (Cheng et al., 1992). These thresholds were all determined objectively by using Lactate‐E software (Newell et al., 2007). As a result of equipment technical issues in the pre‐intervention testing, only 13 participants completed the LT test as described above. These data are reported to *n* = 13 (CO*N* = 6; BFR = 7).

### Exercise training

Participants completed a 6 week supervised SIT intervention, consisting of two training sessions per week, with each session separated by ≥ 48 h. Participants were encouraged to maintain their regular training regime, except for any form of high‐intensity interval training. Each training session consisted of repeated 30 s maximal sprints, from a rolling start, on a mechanically braked cycle ergometer (SE‐780 50; Monark, Stockholm, Sweden) against a manually applied resistance of 0.075 kg·kg body mass^−1^. The training was progressive whereby the first three sessions comprised four repeated sprints, with an additional sprint added on the fourth, seventh and tenth session. Each sprint was separated by a 4.5 min recovery period during which participants immediately dismounted the cycle ergometer and lay in a semi‐supine position on a couch. In the BFR condition, pneumatic pressure cuffs (SC12L; Hokanson, Carmel, IN, USA) were rapidly applied (within 25 s of each sprint) as high up as possible on the proximal portion of each thigh and inflated (E20 Rapid Cuff Inflator and AG101 Cuff Inflator Air Source; Hokanson) to a pressure of 120 mmHg for 2 min (Mitchell et al., 2019). This pressure was kept constant throughout the 6 week training period. The cuffs were then rapidly deflated, and participants remained in the semi‐supine position until 30 s before the next sprint where they re‐mounted the ergometer in time for the subsequent sprints, which began precisely 4.5 min after the previous sprint had ended. In CON, participants remained in the semi‐supine position before re‐mounting the ergometer in time for the next sprint. Measurements of PPO, MPO and MIN were obtained from each sprint (Monark software; Monark). Total work completed throughout the training period was calculated as the cumulative sum of each sprint MPO multiplied by duration of the sprint (30 s).

### Muscle sampling and analysis

Muscle biopsies were obtained at rest from the lateral portion of the vastus lateralis muscle under local anaesthesia (4 mL; 1% lidocaine hydrochloride) using the percutaneous needle biopsy (Bergström, 5 mm diameter; Dixons Surgical Instruments Ltd, Wickford, UK) technique with suction. Pre‐ and post‐training samples were obtained through separate incisions 2 cm apart on the same leg. Approximately 10–20 mg of wet weight (ww) muscle tissue was separated and placed into ice‐cold BioPS solution and stored on ice for high‐resolution respirometry analysis on the same day. The remainder of the sample was immediately snap‐frozen in liquid nitrogen and stored at −80°C until analysis.

### High‐resolution respirometry

Muscle samples were placed in ice cold BioPS [2.77 mm CaK_2_EGTA, 7.23 mm K_2_EGTA, 5.77 mm Na_2_ATP, 6.56 mm MgCl_2_, 20 mm taurine, 50 mm 2‐(*N*‐morpholino) ethanesulfonic acid, 15 mm Na_2_‐phosphocreatine, 20 mm imidazole and 0.5 mm DTT, adjusted to pH 7.1 using titrations of KOH]. Under a low‐power microscope, muscle samples were dissected of connective tissue and fat prior to mechanical separation of two 1–3 mg ww muscle fibre bundles. For chemical permeabilisation of the plasma membrane, the muscle fibres were quickly transferred into a saponin solution [50 µg·mL^−1^ BioPS; 20 µL of saponin stock (5 mg saponin·mL^−1^ BioPS) in 2 mL of BioPS] and gently agitated for 30 min on ice. The samples were then transferred into 2 mL of MiR05 [a respiration medium containing: 110 mm sucrose, 60 mm K^+^‐lactobionate, 0.5 mm EGTA, 3 mm MgCl_2_, 20 mm taurine, 10 mm KH_2_PO_4_, 20 mm 4‐(2‐hydroxyethyl)piperazine‐1‐ethanesulfonic acid (HEPES; adjusted to pH 7.1 with KOH at 37°C) and 1 g·L^−1^ bovine serum albumin essentially fatty acid free] and gently agitated for 10 min on ice to remove any remaining saponin solution from the tissue, before being transferred into a fresh 2 mL of MiR05 (Pesta & Gnaiger, 2012). The muscle fibres were then dried on filter paper, weighed and transferred into a fresh droplet of ice‐cold MiR05. Mitochondrial respiration was measured in duplicate (from 1–3 mg ww of muscle fibres) after fully immersing the fibres into MiR05) at 37°C in the chamber of a high‐resolution respirometer (O2k; Oroboros, Innsbruck, Austria). Using DatLab 7.3 software (Oroboros), the O_2_ concentration of the media (in nmol·mL^−1^) and flux (pmol·s^−1^·mg^−1^) were instantaneously recorded. To avoid any potential O_2_ diffusion limitation, the O_2_ concentration was maintained within the range 250–500 µm (Pesta & Gnaiger, 2012). Reoxygenation by direct syringe injection of pure O_2_ was administered when necessary. The set‐points of O_2_ concentration were fixed based on daily air calibration and high‐to‐zero O_2_ instrumental background calibration of the polygraphic oxygen sensor electrode within each chamber, in accordance with the manufacturer instructions (Oroboros).

Permeabilised muscle fibres were analysed using substrate‐uncoupler‐inhibitor‐titration (SUIT) protocol 8 (DatLab 7.3; Oroboros) with some minor alterations where specified. The SUIT protocol sequence comprised: 10 mm glutamate and 5 mm pyruvate in the absence of adenylates to measure leak respiration (_L_) through mitochondrial protein complex (C) I (CI_L_). To ensure saturating concentrations of ADP, 5 mm was initially titrated, followed by multiple 2.5 mm ADP titrations until respiration plateaued. Subsequently, 0.5 mm malate was added to determine maximum ADP‐stimulated respiration, as an *ex vivo* marker of oxidative phosphorylation (_P_), capacity through CI (CI_P_). Next, 10 mm succinate was added, with further repeated titrations of 2.5 mm ADP until respiration plateaued, to ensure saturation through the succinate linked CII respiratory pathway. The subsequent maximum steady‐state respiration therefore represents _P_ through CI and CII (CI+II_P_). Following this, 10 µm cytochrome *c* was added to test the integrity of the inner mitochondrial membrane, with all samples meeting a less than 15% increase in respiration, deemed acceptable for successful plasma membrane, but not mitochondrial membrane, permeabilisation. A series of stepwise carbonyl cyanide 4‐(trifluoromethoxy) phenylhydrazone (i.e. FCCP) titrations (0.75–1.5 µm) were then added until O_2_ flux plateaued, representing maximal electron transfer system (ETS) capacity (_E_) through CI and CII (CI+II_E_). Afterwards, 0.5 µm rotenone, a CI inhibitor, was added for the determination of _E_ through CII alone (CII_E_). Addition of 2.5 µm antimycin A, a CIII inhibitor, allowed for the determination of residual O_2_ consumption of the electrode, indicative of non‐mitochondrial respiration, for later correction of respiratory measures. Next, artificial electron donors for CIV, 2 mm ascorbate and 0.5 mm *N*,*N*,*N*9,*N*9‐tetramethyl‐*p*‐phenylenediamine (TMPD), were added to measure _E_ through CIV (CIV_E_). Lastly, ≥ 100 mm of sodium azide was added, inhibiting all mitochondrial respiration, to calculate ascorbate/TMPD‐mediated increases in autoxidation of the O2k electrode, aiming to correct CIV_E_. Data are expressed relative to sample ww and CS activity. Internal flux control ratios (FCRs) of CS activity‐corrected mitochondrial respiration were calculated for each time point in each of the experimental conditions. Briefly, the leak control ratio (LCR), the quotient of CI_L_ over CI+II_E_; the phosphorylation control ratio (PCR), the quotient of CI+II_P_ over CI+II_E_; the coupling or inverse respiratory control ratio (InvRCR), the quotient of CI_L_ over CI+II_P_; the substrate control ratio (SCR), the quotient of CI_P_ over CI+II_P_ at constant OXPHOS; and reserve CIV capacity (CIVres), the quotient of CI+II_P_ over CIV_E_. Because of the availability of tissue and technical considerations, high‐resolution respirometry analysis was conducted in 12 participants. These data are reported to *n* = 12 (CON = 6; BFR = 6).

### CS activity assay

Frozen muscle tissue (15–80 mg) was homogenised in cold lysis buffer (1:10, ww:volume) containing PBS, 0.2% Triton X‐100, and protease and phosphatase inhibitor cocktail (Thermo Fisher Scientific, Loughborough, UK). Using a tissue lyser (Qiagen, Manchester, UK), samples were blitzed twice at 20 Hz for 4 min, centrifuged at 12,000 × ***g*** for 10 min to pellet insoluble material and the supernatant was transferred to a fresh Eppendorf tube. The Pierce 660 protein assay was used to determine protein concentrations in accordance with manufacturer's instructions (Thermo Fisher Scientific). Subsequently, CS was analysed on a 96‐well plate. Each well contained 150 µL of 100 mm Tris buffer (pH 8.3), 10 µL of 1 mg·mL^−1 ^muscle homogenate, 25 µL of 1 mm 5,5‐dithio‐bis (2‐nitrobenzoic acid), 40 µL of 3 mm acetyl coenzyme A and 10 µL of 1% Triton X‐100. Rapid, multiwell titration of 15 µL of 10 mm oxaloacetate (pH 8.3) immediately preceded placing the plate into a spectrophotometer (Varioskan Flash; Thermo Fisher Scientific) maintained at 30°C. Following 30 s of linear agitation, absorbance at 412 nm was measured every 15 s for 3 min. Data were corrected for the pathlength of the plate and expressed as mol·h^−1^·kg^−1^. The co‐efficient of variation for this assay was 8.3 ± 6.1%.

### Statistical analysis

The total work completed during the training block and in individual sessions were analysed using an unpaired *t*‐test. Subsequent analyses were performed using two‐factor repeated measures ANOVA with one within‐factor (time; pre *vs*. post) and one between‐factor (condition; CON *vs*. BFR). Where significant effects were observed, *post‐hoc* paired *t*‐tests were used to locate differences. Effect sizes (ES) for *post‐hoc t‐*tests were calculated using Cohen's *d* statistic, interpreted using Hopkins’ (2002) categorisation criteria (small: 0.2–0.59; medium: 0.6–1.19; large: > 1.2). All data are presented as the mean ± SD. *P* < 0.05 was considered statistically significant.

## Results

### Training intervention

All participants completed 100% of the supervised training sessions without any complications. The total work completed was not different (*P* = 0.82) between CON (1178 ± 128 kJ) and BFR (1193 ± 116 kJ) over the 6 week training, nor between any single session (*P* ≥ 0.38).

### CS activity

**Figure 1:**
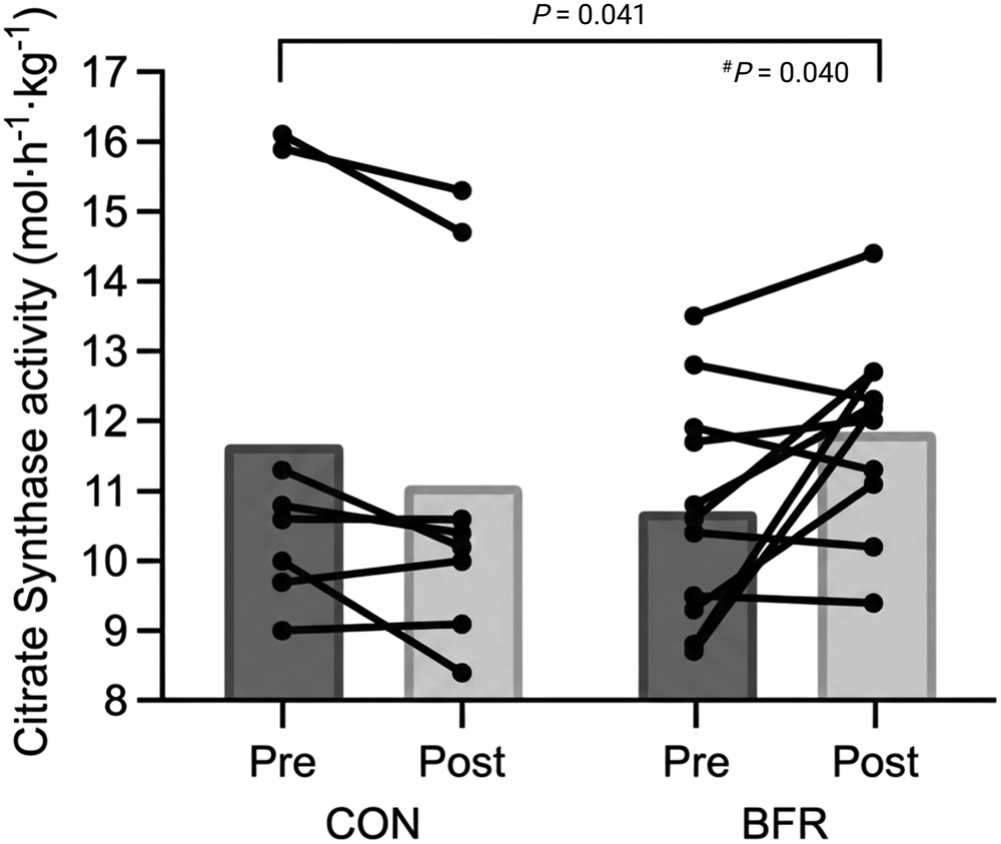
Citrate synthase before and after control (CON) and blood‐flow restriction (BFR) training interventions Bars represent the mean value. Lines represent individual responses. ^§^Time × condition interaction; #*post hoc t* test pre *vs*. post. Data are CON, *n = *8; BFR, *n* = 12.

There was no difference in CS activity (Fig. 1) between groups at baseline (*P = *0.36; ES = 0.21). There was a significant time × condition interaction for CS activity (*P* = 0.011); however, there was no main effect of time (*P = *0.389) or condition (*P* = 0.932). CS activity increased following training in BFR (*P = *0.040; ES = 0.68) but not in CON (*P = *0.053; ES = 0.82). There was, however, no difference in CS activity between CON and BFR post‐training (*P = *0.39; ES = 0.20).

### Mitochondrial respiration

Mass‐specific mitochondrial respiration parameters are presented in Table 1. There were no differences between CON and BFR in any mass‐corrected mitochondrial respiration parameters pre‐training (*P* ≥ 0.135; ES ≤ 0.47). There were main effects of time and time × condition interactions for mass‐corrected CII_E_ and CIV_E_ both of which increased following training in BFR but not in CON. There were main effects of time for mass‐corrected CI_P_ and CI+II_P_ both of which increased following training in BFR, but not in CON. There was a main effect of time for mass‐corrected CI+II_E_, which increased following training in in CON but not in BFR.

**Table 1:** Mass‐corrected mitochondrial respiration parameters before and after control (CON) and blood‐flow restriction (BFR) training interventions

pmol·s^−1^·mg^−1^	CON					BFR					ANOVA interaction (*P*)	ANOVA time effect (*P*)	ANOVA condition effect (*P*)
Parameter	Pre	Post	% change	*Post‐hoc t*‐test (*P*)	Effect size (*d*)	Pre	Post	% change	*Post‐hoc t*‐test (*P*)	Effect size (*d*)			
**CI_L_**	3.0 ± 1.2	5.6 ± 2.7	102 ± 142	ND	0.80	3.5 ± 1.9	4.1 ± 3.5	5 ± 54	ND	0.25	0.327	0.092	0.834
**CI_P_**	46.4 ± 10.7	51.3 ± 13.7	13 ± 30	0.342	0.43	42.1 ± 14.6	64.5 ± 21.4	65 ± 59	**0.031**	1.22	0.078	**0.012**	0.581
**CI+II_P_**	71.3 ± 9.6	82.7 ± 15.1	17 ± 18	0.086	0.87	70.4 ± 24.9	108.1 ± 32.4	72 ± 73	**0.050**	1.05	0.122	**0.011**	0.258
**CI+II_E_**	80.1 ± 13.2	94.1 ± 20.0	19 ± 17	**0.027**	1.26	82.7 ± 27.1	123.0 ± 43.1	61 ± 65	0.059	0.99	0.156	**0.010**	0.250
**CII_E_**	29.3 ± 5.6	32.2 ± 15.7	8 ± 28	0.471	0.32	30.3 ± 7.2	51.6 ± 12.4	77 ± 51	**0.007**	1.82	**0.012**	**0.003**	0.065
**CIV_E_**	173.7 ± 48.0	163.8 ± 61.3	−2 ± 42	0.731	0.15	140.3 ± 15.1	236.1 ± 37.3	70 ± 34	**0.002**	2.38	**0.008**	**0.023**	0.345

*Note*: Values are the mean ± SD. Data are CON, *n* = 6; BFR, *n* = 6.

Abbreviations: CI_L_, leak respiration through mitochondrial protein complex (C) I of the electron transfer system (ETS); CI_P_ and CI+II_P_, ADP‐stimulated respiration through CI and CII of the ETS, respectively; CI+II_E_, CII_E_ and CIV_E_, maximal non‐coupled ETS capacity of CI and CII, CII, and CIV, respectively. ND, not determined. Significance denoted in bold.

Mitochondrial respiration parameters expressed relative to CS activity (CS activity‐corrected) are presented in Table 2. There were no differences between CON and BFR in any CS activity‐corrected mitochondrial respiration parameters pre‐training (*P* ≥ 0.285; ES ≤ 0.46). There were main effects of time and time × condition interactions for CII_E_ and CIV_E_ both of which increased following training in BFR but not in CON. Furthermore, CS activity‐corrected CII_E_ was greater following training in BFR compared with CON. There was a main effect of time for CS activity‐corrected CI_P_, which increased following training in BFR but not CON. There was a main effect of time for CS‐corrected CI+II_P_; however, *post‐hoc* paired *t‐*tests revealed no changes in BFR or CON. There was a main effect of time for CS activity‐corrected CI+II_E_, which increased following training in CON but not in BFR.

**Table 2:** Citrate synthase activity (CS)‐corrected mitochondrial respiration parameters before and after control (CON) and blood‐flow restriction (BFR) training interventions

pmol·s^−1^·CS^−1^	CON					BFR					ANOVA interaction (*P*)	ANOVA time effect (*P*)	ANOVA condition effect (*P*)
Parameter	Pre	Post	% change	*Post‐hoc t*‐test (*P*)	Effect size (*d*)	Pre	Post	% change	*Post‐hoc t*‐test (*P*)	Effect size (*d*)			
**CI_L_**	0.25 ± 0.09	0.43 ± 0.23	114 ± 151	ND	0.71	0.30 ± 0.18	0.34 ± 0.27	21 ± 70	ND	0.27	0.219	0.126	0.779
**CI_P_**	3.90 ± 1.10	4.41 ± 0.94	19 ± 36	0.326	0.44	3.63 ± 1.40	5.28 ± 2.12	66 ± 62	**0.049**	1.06	0.235	**0.037**	0.680
**CI+ II_P_**	6.03 ± 1.45	7.10 ± 0.55	22 ± 22	0.107	0.80	6.09 ± 2.47	8.82 ± 3.23	77 ± 79	0.081	0.89	0.314	**0.035**	0.387
**CI+II_E_**	6.74 ± 1.58	8.19 ± 0.99	25 ± 21	**0.038**	1.14	7.12 ± 2.62	10.05 ± 4.19	64 ± 69	0.084	0.88	0.410	**0.029**	0.395
**CII_E_**	2.47 ± 0.65	2.71 ± 0.70	13 ± 34	0.460	0.33	2.59 ± 0.76	4.19 ± 1.27	83 ± 64	**0.030**	1.23	**0.047**	**0.012**	0.080
**CIV_E_**	15.31 ± 6.45	14.17 ± 5.73	3 ± 45	0.664	0.19	11.88 ± 1.61	19.53 ± 2.78	68 ± 37	**0.002**	2.41	**0.010**	**0.041**	0.704

*Note*: Values are the mean ± SD. Data are CON, *n* = 6; BFR, *n* = 6.

Abbreviations: CI_L_, leak respiration through mitochondrial protein complex (C) I of the electron transfer system (ETS); CI_P_ and CI+II_P_, ADP‐stimulated respiration through CI and CII of the ETS, respectively; CI+II_E_, CII_E_, and CIV_E_, maximal non‐coupled ETS capacity of CI and CII, CII and CIV, respectively. ND, not determined. Significance denoted in bold.

Internal FCR are presented in Table 3. PCR and InvRCR were different at baseline between groups (*P = *0.047; ES = 0.825 and *P = *0.026; ES = 0.021). There were no other differences between CON and BFR pre‐training. There was a time × condition interaction effect for InvRCR; however, *post‐hoc* paired *t*‐tests did not detect changes over time in either CON or BFR (Table 3), nor between groups following training (*P = *0.781; ES = 0.604). No further interaction, time or condition effects were observed.

**Table 3:** Citrate synthase activity (CS)‐corrected mitochondrial respiration flux control ratios before and after control (CON) and blood‐flow restriction (BFR) training interventions

Flux control ratios	CON					BFR					ANOVA interaction (*P*)	ANOVA time effect (*P*)	ANOVA condition effect (*P*)
Parameter	Pre	Post	% change	*Post‐hoc t*‐test (*P*)	Effect size (*d*)	Pre	Post	% change	*Post‐hoc test* (*P*)	Effect size (*d*)			
**LCR**	0.04 ± 0.01	0.05 ± 0.03	41.4	ND	0.598	0.04 ± 0.02	0.04 ± 0.03	−18.2	ND	0.275	0.571	0.574	0.159
**PCR**	0.89 ± 0.04	0.87 ± 0.08	−2.2	ND	0.200	0.84 ± 0.08	0.89 ± 0.05	5.7	ND	1.058	0.613	0.602	0.184
**Inv‐RCR**	0.11 ± 0.05	0.06 ± 0.03	−44.0	0.068	1.101	0.11 ± 0.10	0.04 ± 0.03	−63.0	0.141	0.595	**0.045**	0.717	0.666
**SCR**	0.65 ± 0.09	0.63 ± 0.15	−2.7	ND	0.144	0.60 ± 0.08	0.59 ± 0.04	−1.5	ND	0.085	0.692	0.438	0.870
**CIVres**	0.43 ± 0.12	0.57 ± 0.23	32.8	ND	0.599	0.50 ± 0.17	0.48 ± 0.22	−4.4	ND	0.100	0.420	0.901	0.268

Abbreviations: CIVres, reserve capacity of mitochondrial protein complex IV, the quotient of CI+II_P_ over CIV_E_; InvRCR, inverse respiratory control ratio the quotient of CI_L_ over CI+II_P_; LCR, leak control ratio, the quotient of leak respiration (CI_L_) over uncoupled electron transfer system capacity (_E_) of CI and CII (CI+II_E_); PCR, phosphorylation control ratio, the quotient of ADP‐stimulated respiration (_P_) through CI and CII of the electron transfer system (CI+II_P_) over CI+II_E_; SCR, substrate control ratio, the quotient of CI_P_ over CI+II_P_ at constant oxidativephosphorylation. ND, not determined. Sifnificance denoted in bold.

### Physiological and performance measures

**Figure 2:**
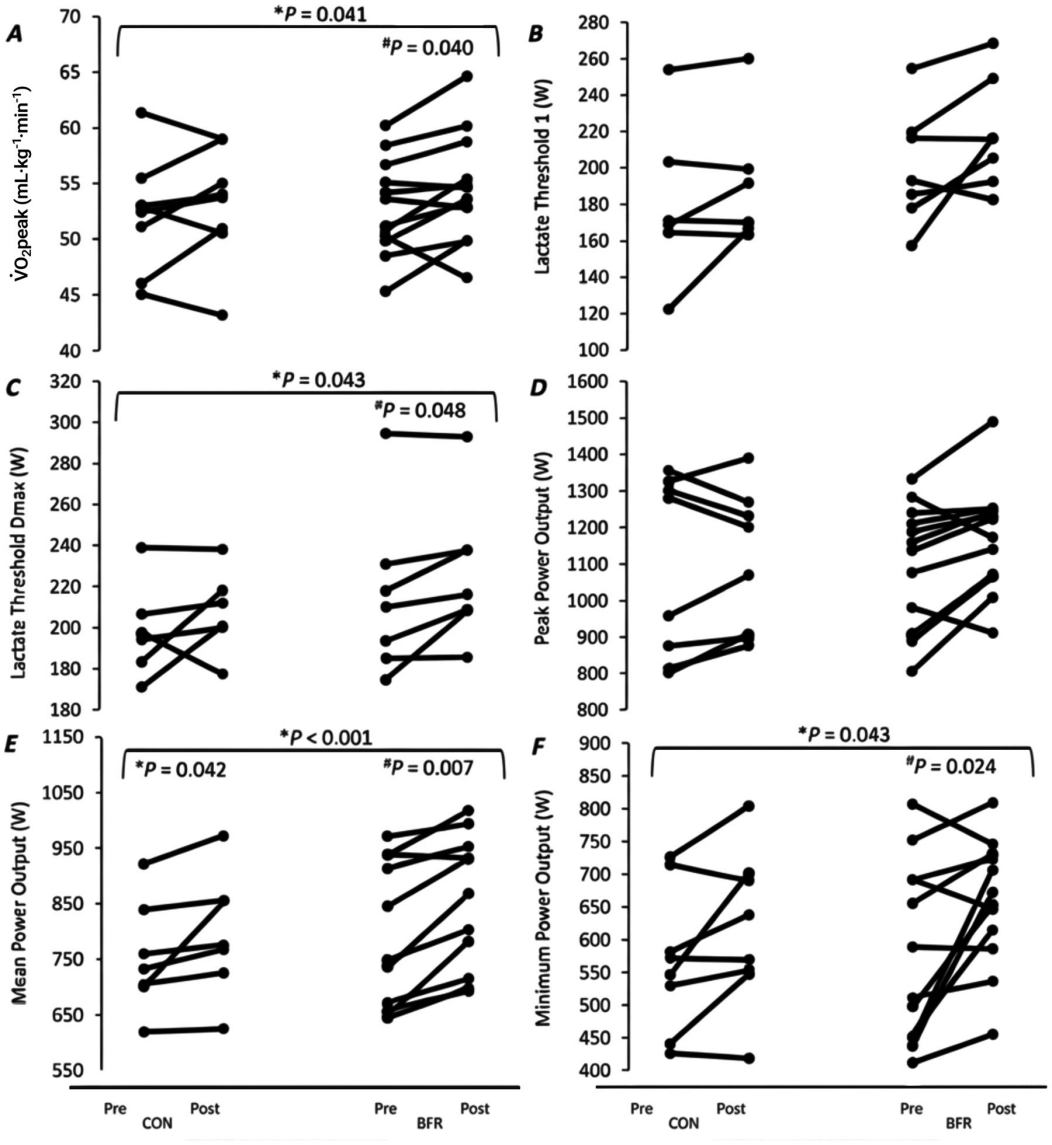
Physiological and performance measures before and after control (CON) and blood‐flow restriction (BFR) training interventions (*A*) ${\dot{V}}_{O_{2}\mathrm{peak}}$; (*B*), 1mmol·L^−1^ increase in blood lacate (B[La]) determined during an incremental step test; (*C*), Dmax maximum perpindular distance from B[La], and line between highest and lowest B[La] determined during incremental step test; (D), Peak power output during 20 s sprint test; (*E*), Mean power output during 20 s sprint test; (*F*) and MIN. Lines represent individual responses. *Main effect of time; ^#^ *post‐hoc* paired *t*‐test pre *vs*. post (*P* < 0.05). Data are CON, *n* = 8, except lactate thresholds, where *n* = 6. BFR, *n* = 12, except lactate thresholds where *n* = 7.

Physiological and performance measures are presented in Table 4. There were no differences between CON and BFR in any physiological and performance measures pre‐training (*P* ≥ 0.250). There were no time × condition interactions or main effects of condition for any of the physiological and performance measures. There were main effects of time for relative ${\dot{V}}_{O_{2}\mathrm{peak}}$, MAP, Dmax, FBLA_4_, MPO and MIN (Fig. 2). *Post‐hoc* paired *t*‐tests revealed increases following training in MAP and MPO in both CON and BFR, whereas ${\dot{V}}_{O_{2}\mathrm{peak}}$, Dmax and MIN only increased following training in BFR.

**Table 4:** Physiological and performance measures before and after control (CON) and blood‐flow restriction (BFR) training interventions

	CON					BFR							
Parameter	Pre	Post	% change	*Post‐hoc t*‐test (*P*)	Effect size (*d*)	Pre	Post	% change	*Post‐hoc t*‐test (*P*)	Effect size (*d*)	ANOVA interaction (*P*)	ANOVA time effect (*P*)	ANOVA condition effect (*P*)
${\dot{V}}_{O_{2}\mathrm{peak}}$ **(L**·**min^−1^)**	4.02 ± 0.38	4.11 ± 0.28	2.5 ± 6.1	ND	0.38	4.26 ± 0.54	4.31 ± 0.51	1.3 ± 4.5	ND	0.26	0.701	0.164	0.297
${\dot{V}}_{O_{2}\mathrm{peak}}$ **(mL**·**min^−1^** ·**kg^−1^)**	52.1 ± 5.2	53.1 ± 5.1	2.1 ± 5.9	0.358	0.35	52.8 ± 4.3	54.5 ± 4.9	3.2 ± 4.9	**0.040**	0.67	0.602	**0.041**	0.64
**MAP (W)**	365 ± 28	391 ± 21	7.4 ± 6.8	**0.039**	0.98	378 ± 48	389 ± 46	3.0 ± 3.8	**0.020**	0.78	0.101	**<0.001**	0.819
**LT 1 mmol·L^−1^ (W)**	177 ± 54	178 ± 46	2.1 ± 20.1	0.922	0.04	199 ± 36	218 ± 34	10.8 ± 14.1	0.071	0.08	0.177	0.227	0.176
**LT Dmax (W)**	198 ± 27	208 ± 20	5.4 ± 11.1	0.305	0.47	215 ± 39	227 ± 34	6.0 ± 6.8	**0.048**	0.88	0.832	**0.043**	0.311
**LT FBLA_4_ (W)**	219 ± 61	236 ± 36	10.4 ± 15.1	0.153	0.69	242 ± 39	264 ± 30	10.5 ± 14.3	0.078	0.75	0.786	**0.022**	0.261
**PPO (W)**	1088 ± 249	1104 ± 197	2.7 ± 8.1	ND	0.19	1098 ± 170	1170 ± 149	7.4 ± 10.2	ND	0.74	0.200	0.051	0.649
**MPO (W)**	745 ± 95	809 ± 108	8.8 ± 10.5	**0.042**	0.87	800 ± 128	852 ± 115	7.0 ± 7.4	**0.007**	0.95	0.729	**<0.001**	0.344
**MIN (W)**	566 ± 110	616 ± 119	9.2 ± 12.1	0.063	0.78	578 ± 137	656 ± 98	17.1 ± 22.5	**0.024**	0.75	0.491	**0.006**	0.605

*Note*: Values are the mean ± SD. Data are CON, *n* = 8, except lactate thresholds where *n* = 6. BFR, *n* = 12, except lactate thresholds where *n* = 7.

Abbreviations: MAP, maximum aerobic power; MIN, minimum power output; MPO, mean power output; PPO, peak power output; ${\dot{V}}_{O_{2}\mathrm{peak}}$, peak oxygen uptake; LT, Lactate threshold; LT 1 mmol·L^−1^, 1 mmol·L^−1^ increase from baseline blood lactate (B[La]); Dmax, maximum perpindular distance from B[La] and line between highest and lowest B[La] determined during incremental step test; FBLA_4_, fixed B[La] of 4 mmol·L^−1^; ND, not determined. Significance denoted in bold.

## Discussion

The main findings of the present study are that: (i) SIT with post‐exercise BFR increased CS activity, implying an increase in mitochondrial content compared to SIT alone; (ii) SIT with post‐exercise BFR augmented the increase in some, but not all, mitochondrial respiratory variables, specifically mass‐ and CS activity‐corrected CII_E_ and CIV_E_; and (iii) the improvements in performance determinants in response to SIT across both groups did not differ between BFR and CON.

This study demonstrated that a 6 week SIT intervention with post‐exercise BFR increased CS activity. Previous research investigating the effect of training with BFR on mitochondrial adaptations did not observe any differences mitochondrial respiratory capacity (Lavigne et al., 2026) following 6 weeks of BFR applied during knee extensor exercise, compared with a knee extensor interval only control limb. Moreover, no changes in CS, COXII and COXIV protein content were observed following 4 weeks of SIT with post‐exercise BFR (Mitchell et al., 2019). These findings are not unexpected given differences in methodologies and muscle analysis. The study by Lavigne et al. (2026) applied BFR during exercise, work‐matching the control limb to the achievable intensity of the BFR limb, limiting the exercise stimulus. Furthermore, mitochondrial respiratory capacity (influenced by both mitochondrial content and respiratory function) was indirectly estimated using near‐infrared spectroscopy (Lavigne et al. 2026). The study by Mitchell et al. (2019) did not limit exercise intensity, utilising post‐exercise SIT with BFR; however, the assessment of mitochondrial protein content, demonstrated to be poorly associated with mitochondrial content, may result in the lack of change in BFR. By contrast, CS activity is more strongly associated with the gold‐standard transmission electron microscopy assessment of mitochondrial content (Larsen et al., 2012). In the present study, CS activity increased following training in BFR, with no change in CON. Despite these observations, the use of CS activity to determine changes in mitochondrial content has been debated. For example, Meinild Lundby et al. (2018) demonstrated that increases in CS activity (44%) did not correlate with changes in mitochondrial content (55%), following 6 weeks of submaximal endurance training. However, CS activity was correlated with mitochondrial density at baseline and post‐training and, accordingly, it was concluded that CS activity may be appropriate to track training‐induced changes in mitochondrial content (volume density), such as the present study. Nevertheless, caution should be exercised when assessing a single mitochondrial protein as a biomarker of mitochondrial content (Groennebaek et al., 2020), with changes in CS activity representative of mitochondrial matrix volume, rather than content specifically, because single protein biomarkers do not differentiate between mitochondrial enlargement and *de novo* biogenesis (Meinild Lundby et al., 2018). The lack of change in CS activity in CON is consistent with research using a similar progressive SIT training intervention over 4 weeks (${\dot{V}}_{O_{2}\mathrm{peak}}$ ∼46 mL·min^−1^·kg^−1^) (Granta et al., 2016), but contrasts with increases in this marker of mitochondrial content following 12 weeks SIT in less trained individuals (${\dot{V}}_{O_{2}\mathrm{peak}}$ ∼33 mL·min^−1^·kg^−1^) (Gillen et al., 2016). The moderately trained (${\dot{V}}_{O_{2}\mathrm{peak}}$: 52.5 ± 4.6 mL·min^−1^·kg^−1^) and regularly physically active participants in the present study are more comparable to the study of Granta et al. (2016) and the lack of increase in CS activity with SIT is consistent with the hypothesis that exercise‐induced changes in mitochondrial content are largely determined by the initial fitness level and both training volume and intensity (Mølmen et al., 2025). Accordingly, the results of the present study suggest that the extension or augmentation of the exercise stimulus, and the resulting metabolic millieu, arising from post‐exercise BFR contributes to the increased CS acitivity, and, by inference, increased mitochondrial content.

This is the first study to demonstrate that SIT combined with BFR can augment improvements in mitochondrial respiration compared to SIT alone. Specifically, maximal uncoupled respiration (CII_E_ and CIV_E_), normalised to both mass and CS activity, increased following training in BFR compared to no change in CON. These data support the hypothesis that mitochondrial structure and functional characteristics, may adapt differentially (Meinild Lundby et al., 2018). SIT has previously been demonstrated to increase parameters of mitochondrial respiration, after 4 weeks of training in a cohort with a baseline ${\dot{V}}_{O_{2}\mathrm{peak}}$ of ∼46 mL·min^−1^·kg^−1^ (Granata et al., 2016). Previous research has observed no increases in oxidative phosphorylation following 6 week cycling interval training with BFR applied during exercise (Christiansen et al., 2020). However, this application of BFR to exercise is demonstrated to significantly limit exercise intensity, which is reported to be essential for adaptations to mitochondrial respiration (Bishop et al., 2025). Additionally, although there were no interaction effects, CI_P_, CI+II_P_ and CI+II_E_ increased following training in both conditions (as indicated by the main effect for time). When considering *post‐hoc* paired *t*‐tests, effect sizes and percentage changes, there is a seemingly consistent pattern of response indicating marginal additional benefits of BFR. Although these do not reach statistical significance [probably as a result of the limited sample size (CON, *n* = 6 *vs*. BFR, *n = *6)], the increases in uncoupled respiration demonstrated by interaction effects and post‐analysis, and this potential pattern of response in coupled respiration, suggest that BFR can be deemed a potent stimulus for augmenting SIT‐induced increases in mitochondrial respiration. Despite no changes in CS activity, increases of ∼25% in maximal coupled and uncoupled respiration have previously been observed following 4 weeks of similarly progressive SIT (Granta et al., 2016), which is comparable to lack of changes in CS activity, and the increases maximal coupled and uncoupled respiration in CON (∼20%) in the present study. In comparison, the increases in maximal coupled and uncoupled respiration with BFR in the present study were ∼50%. This, combined with the increases in CS activity of ∼12%, further highlights the potency of SIT combined with BFR stimulus to augment mitochondrial adaptation, through improvements in both mitochondrial respiration and content, compared with SIT alone.

The lack of changes in FCR, concomitant with increases in mitochondrial respiratory parameters following training in the present study, is in line with previous research (Meinild Lundby et al., 2018). The findings of the present study are perhaps unsurprising, given the pattern of response for coupled and uncoupled respiration to increase following training. The maintenance of mitochondrial coupling efficiency alongside increases in the overall capacity of the ETS further indicates that, following training, participants are more capable of meeting high ATP flux demands. Moreover, these findings may indicate that improvements in mitochondrial respiration occur through morphological changes, as a result of the maintenance of internal control ratios. The baseline differences between groups in both PCR and InvRCR, are unexpected, given that these were not reflected in absolute values for mass‐ or CS‐activity specific mitochondrial respiration. Because of the variability of respirometry measures, particularly when comparing between biopsies, in different individuals, taken at multiple time points (Kuang et al., 2022), it is important to interpret mitochondrial respirometry normalisation data together to best draw conclusions. Therefore, despite the differences in these baseline measures, the evidence from the within group comparisons of FCR, alongside the changes in CS activity and mitochondrial respiration, support the conclusion that SIT with post‐exercise BFR can induce functional changes in mitochondrial respiratory capacity.

BFR has been purported to augment the activation of signalling pathways of mitochondrial biogenesis beyond that observed with exercise alone (Ferguson et al, 2021), although the evidence is contradictory. For example, low‐intensity running combined with BFR augmented the increase in AMPK activation and PGC‐1α mRNA expression compared to low‐intensity exercise alone (Christiansen et al., 2018). By contrast, there were no differences in AMPK activation or PGC‐1α mRNA expression following sprint interval exercise with post‐exercise BFR (Taylor et al., 2016). However, it has been suggested that training‐induced changes in mitochondrial respiration may be mediated by different molecular pathways or time courses than those considered for mitochondrial content. Recent research has demonstrated that sprint interval exercise, but not moderate‐intensity exercise, elicits acute mitochondrial ultrastructural and morphological disturbances, consistent with mitochondrial swelling, damage and subsequent mitophagy, and concomitant with greater alterations in transcriptomics associated with integrated and mitochondrial stress responses (Botella et al., 2025). Additionally, the p53 pathway has emerged as exerting transcriptional control of mitochondrial and respiratory genes (Granata et al., 2016; Granata et al., 2018). Activation of p53 is associated with increased mitochondrial respiration (Matoba et al., 2006), content (Saleem et al., 2011), structure (Sevrioukova, 2011), and the assembly, activity and stability of the ETS (Sevrioukova, 2011; Vahsen et al., 2004). The increased mitochondrial content and respiration with BFR in the present study is consistent with the hypothesis that the p53 pathway is stimulated by the physiological stressors arising from SIT, augmented by BFR. This is especially true given the lack of changes in CS activity with SIT, despite changes in mitochondrial respiratory rates and upregulation of mitochondrial stress in response to 8 weeks of SIT (Botella et al., 2025). Moreover, the increased p53 in response to SIT (Granta et al. 2016) may explain the dissociation between changes in mitochondrial respiration and content in CON in the present study. p53 is demonstrated to regulate autophagy (Maiuri et al., 2010; Saleem et al., 2009), fission (Li et al., 2010) and fusion (Wang et al., 2010), where mitochondria undergo ‘repair’. This incorporation of newly synthesised proteins to replace damaged ones may enhance mitochondrial function independent of changes to mitochondrial content (Johnson et al., 2013; Mai et al., 2012). Moreover, p53 interacts with mitochondrial transcription factor A (TFAM) (Park et al., 2009; Saleem & Hood, 2013; Yoshida et al., 2003), a transcription factor that regulates the transcription of mtDNA‐encoded subunits of the ETS, important in exercise‐induced mitochondrial biogenesis (Scarpulla, 2011). Significantly, TFAM mRNA has been observed to be upregulated only in response to exercise of higher intensities (Popov et al., 2014). Either way, a greater understanding of the mechanisms by which exercise intensity and BFR induce mitochondrial adaptations, particularly in trained individuals is required.

The potency of SIT for increasing ${\dot{V}}_{O_{2}\max}$ and markers of endurance and sprint performance is well‐established (Behringher et al., 2017; Gist et al. 2014; Sloth et al. 2013; Yang et al., 2025). However, in trained cohorts, SIT alone is inadequate to induce performance improvements (Mitchell et al., 2019). In the present study, there were no significant interactions for any of the physiological markers of endurance performance, although main effects of time were observed for ${\dot{V}}_{O_{2}\mathrm{peak}}$ (relative), MAP and LT (Dmax and FBLA_4_), suggesting an increase in these endurance performance markers in both CON and BFR groups. *Post‐hoc* paired *t*‐tests, however, revealed that ${\dot{V}}_{O_{2}\mathrm{peak}}$ (relative) and LT (Dmax) increased compared to baseline (with moderate effect sizes) following BFR. However, comparison between groups for ${\dot{V}}_{O_{2}\mathrm{peak}}$ data must be treated with caution because of the unbalanced groups for this parameter. Additionally, the lack of confirmation test for maximal oxygen uptake should also be considered when interpreting the results of the present study. Although participants met secondary criteria for the attainment of ${\dot{V}}_{O_{2}\mathrm{peak}}$ (Poole et al., 2016), ${\dot{V}}_{O_{2}\max}$ was not confirmed by a verification test, due to the potential impact of sprint testing performed on the same visit and aims to prevent undue impact on subsequent exercise testing. Consideration of the surprising reductions in markers of endurance performance in some individuals in this study (Fig. 2) may indicate non‐functional overreaching in some participants (Meeusen et al., 2006). The SIT intervention (12 sessions, 66 maximal efforts) coupled with the maintenance of normal training may have hampered performance in exercise tests, despite reasonable precautions to avoid this (at least 4 days between final training session and testing). In a real‐world application, an intensive training block to improve markers of endurance performance such as this would be followed by a considerable ‘taper’ to ensure optimal endurance exercise performance (Kubukeli et al., 2002). However, examination of the primary outcome measure of mitochondrial biogenesis, with minimal delay and influence of extraneous variables, prevented further rest or workload reduction.

In conclusion, BFR augmented the increase in mitochondrial content and some mitochondrial respiratory variables compared to SIT alone. Despite these improved mitochondrial adaptations with BFR, there was no clear benefit to any physiological or performance markers compared to SIT alone.

## Additional information

## Competing interests

The authors declare that they have no competing interests.

## Author contributions

D.L.P., C.S. and R.A.F. conceived and designed research. D.L.P., C.S. and R.A.F. performed experiments. D.L.P. and C.S. analysed data. D.L.P., C.S., E.A.M., S.J.B. and R.A.F. interpreted results of experiments. D.L.P. prepared figures. D.L.P., and R.A.F. drafted manuscript. D.L.P., S.J.B. and R.A.F. edited and revised manuscript. D.L.P., C.S., E.A.M., S.J.B. and R.A.F. approved the final version of the manuscript submitted for publication.

## Funding

None.

## Supporting information


Peer Review History

## Data Availability

The raw data supporting the conclusions of this research is available from the corresponding author upon reasonable request.
