# Supplementary material for: Sprint‐interval training with post‐exercise blood flow restriction increases mitochondrial content and respiration
Source: J Physiol. 2026 Jun 3;604(14):5968–83. doi: 10.1113/JP290955 (PMC13370709; doi:10.1113/JP290955)
Supplement: Supplementary file 1 — Peer Review History [file TJP-604-5968-s001.pdf]

The Journal of Physiology JP

Sprint-Interval Training with Post-Exercise Blood Flow Restriction Increases Mitochondrial Content and Respiration

Donald Lang Peden, Carolin Stangier, Emma Mitchell, Stephen J Bailey, and Richard A. Ferguson
DOI: 10.1113/JP290955

Corresponding author(s): Donald Peden (donald.peden@nottingham.ac.uk)

The referees have opted to remain anonymous.

Review Timeline:

Submission Date:	16-Jan-2026
Editorial Decision:	24-Feb-2026
Revision Received:	16-Apr-2026
Editorial Decision:	21-Apr-2026
Revision Received:	23-Apr-2026
Accepted:	06-May-2026

Senior Editor: Karyn Hamilton

Reviewing Editor: Robert Musci

Transaction Report:

(Note: With the exception of the correction of typographical or spelling errors that could be a source of ambiguity, letters and reports are not edited. Depending on transfer agreements, referee reports obtained elsewhere may or may not be included in this compilation. Referee reports are anonymous unless the Referee chooses to sign their reports.)

Re: JP-RP-2026-290955 "**Sprint-Interval Training with Post-Exercise Blood Flow Restriction Increases Mitochondrial Content and Respiration**" by Donald Lang Peden, Carolin Stangier, Emma Mitchell, Stephen J Bailey, and Richard A. Ferguson

Dear Dr Peden,

Thank you for submitting your manuscript to The Journal of Physiology. It has been assessed by a Reviewing Editor and by 2 expert referees and we are pleased to tell you that it is potentially acceptable for publication following satisfactory major revision.

Please advise your co-authors of this decision as soon as possible.

The referee reports are copied at the end of this email.

Please address all the points raised and incorporate all requested revisions or explain in your Response to Referees why a change has not been made. We hope you will find the comments helpful and that you will be able to return your revised manuscript within 2 months. If your article is NOT for a Special Issue, you may have 9 months to revise. If you require an extension, please contact journal staff: jp@physoc.org. Please note that this letter does not constitute a guarantee for acceptance of your revised manuscript.

Your revised manuscript should be submitted online using the link in your Author Tasks: Link Not Available. This link is accessible via your account as Corresponding Author; it is not available to your co-authors. If this presents a problem, please contact journal staff (jp@physoc.org). Image files from the previous version are retained on the system. Please ensure you replace or remove any files that are being revised.

If you do not wish to submit a revised version of your manuscript, you must inform our journal staff (jp@physoc.org) or reply to this email to request withdrawal. Please note that a manuscript must be formally withdrawn from the peer review process at one journal before it may be submitted to another journal.

TRANSPARENT PEER REVIEW POLICY: To improve the transparency of its peer review process, The Journal of Physiology publishes online as supporting information the peer review history of all articles accepted for publication. Readers will have access to decision letters, including Editors' comments and referee reports, for each version of the manuscript, as well as any author responses to peer review comments. Referees can decide whether or not they wish to be named on the peer review history document.

ABSTRACT FIGURES: Authors are expected to use The Journal's premium BioRender account to create/redraw their Abstract Figures. Information on how to access this account is here: https://physoc.onlinelibrary.wiley.com/journal/14697793/biorender-access.

This will enable Authors to create and download high-resolution figures. If authors have used the free BioRender service, they can use the instructions provided in the link above to download a high-resolution version suitable for publication.

The link provided should only be used for the purposes of this submission. Authors will be charged for figures created on this account if they are not related to this manuscript submission.

LANGUAGE EDITING AND SUPPORT FOR PUBLICATION: If you would like help with English language editing, or other article preparation support, Wiley Editing Services offers expert help, including English Language Editing, as well as translation, manuscript formatting, and figure formatting at www.wileyauthors.com/eeo/preparation. You can also find resources for Preparing Your Article for general guidance about writing and preparing your manuscript at www.wileyauthors.com/eeo/prepresources.

RIGOUR AND REPRODUCIBILITY: We recommend authors consult The Journal's Rigour and Reproducibility requirements to ensure that their manuscript is suitable for publication: https://physoc.onlinelibrary.wiley.com/pb-assets/hub-assets/physoc/documents/TJP-Rigour-and-Reproducibility-Requirements.pdf

REVISION CHECKLIST:

Check that your Methods section conforms to journal policy: https://jp.msubmit.net/cgi-bin/main.plex?form_type=display_requirements#methods.

Check that data presented conforms to the statistics policy: https://jp.msubmit.net/cgi-bin/main.plex?form_type=display_requirements#statistics.

Upload a full Response to Referees file. To create your 'Response to Referees': copy all the reports, including any comments from the Senior and Reviewing Editors, into a Microsoft Word, or similar, file and respond to each point, using font or background colour to distinguish comments and responses and upload as the required file type.

Please upload two versions of your manuscript text: one with all relevant changes highlighted and one clean version with no changes tracked. The manuscript file should include all tables and figure legends, but each figure/graph should be uploaded as separate, high-resolution files.

You may also upload:

- 'Potential Cover Art' for consideration as the issue's cover image
- Appropriate Supporting Information (Video, audio or data set: see https://jp.msubmit.net/cgi-bin/main.plex?form_type=display_requirements#supp).

We look forward to receiving your revised submission.

If you have any queries, please reply to this email and we will be pleased to advise.

Yours sincerely,

Karyn Hamilton
Senior Editor
The Journal of Physiology

REQUIRED ITEMS

- Author photo and profile. First or joint first authors are asked to provide a short biography (no more than 100 words for one author or 150 words in total for joint first authors) and a portrait photograph. These should be uploaded and clearly labelled together in a Word document with the revised version of the manuscript. See Information for Authors for further details.

- Papers must comply with the Statistics Policy: https://jp.msubmit.net/cgi-bin/main.plex?form_type=display_requirements#statistics.

In summary:

- If n {less than or equal to} 30, all data points must be plotted in the figure in a way that reveals their range and distribution. A bar graph with data points overlaid, a box and whisker plot or a violin plot (preferably with data points included) are acceptable formats.

- If n > 30, then the entire raw dataset must be made available either as supporting information, or hosted on a not-for-profit repository, e.g. FigShare, with access details provided in the manuscript.

- 'n' clearly defined (e.g. x cells from y slices in z animals) in the Methods. Authors should be mindful of pseudoreplication.

- All relevant 'n' values must be clearly stated in the main text, figures and tables.

- The most appropriate summary statistic (e.g. mean or median and standard deviation) must be used. Standard Error of the Mean (SEM) alone is not permitted.

- Exact p values must be stated. Authors must not use 'greater than' or 'less than'. Exact p values must be stated to three significant figures even when 'no statistical significance' is claimed.

- Please include an Abstract Figure file and an Abstract Figure legend. An appropriate figure legend, which should not exceed 150 words in length, should be included in the main manuscript file. The Abstract Figure is a piece of artwork designed to give readers an immediate understanding of the research and should summarise the main conclusions. If possible, the image should be easily 'readable' from left to right or top to bottom. It should show the physiological relevance of the manuscript so readers can assess the importance and content of its findings. Abstract Figures should not merely recapitulate other figures in the manuscript. Please try to keep the diagram as simple as possible and without superfluous information that may distract from the main conclusion(s). Abstract Figures must be provided by authors no later than the revised manuscript stage and should be uploaded as a separate file during online submission labelled as File Type 'Abstract Figure'. Please also ensure that you include the figure legend in the main article file. All Abstract Figures should be created using BioRender. Authors should use The Journal's premium BioRender account to export high-resolution images. Details on how to use and access the premium account are included as part of this email.

EDITOR COMMENTS

Reviewing Editor:
Thank you for your submission. Both referees highlighted the importance of your investigation and the methodological approach employed. Both referees had salient suggestions in improving the manuscript and highlighted a couple weaknesses. Please address all of them.

Please pay particular attention the valid concerns raised regarding the imbalance sample sizes, post-hoc analyses, and interpretation of the interaction between time and training modality. If the authors believe that the imbalance between intervention groups does not influence the results, they should provide a robust counter-argument to these concerns.

The authors use CS activity as a means of estimating mitochondrial content. While it is appreciated that in the past it has been used a valid marker of mitochondrial content in a limited cohort of individuals (PMID: 22586215), more recent investigations raise cause for concern (r=0460, PMID: 32255679) and (r=0.546, PMID: 28580772). Additionally, a change in CS activity fails to correlate/predict changes in mitochondrial volume (PMID: 28580772) particularly after exercise training. Given recent data, it seems inappropriate to use CS activity as a marker of content and/or use it as a means to evaluate intrinsic mitochondrial capacity.

Minor comments/ considerations:

The authors should also consider to expand their analysis of their respirometry data by evaluating flux control ratios (P/E, L/E, L/P).

The authors presumably use "ES" as an abbreviation for "Effect Size" in the results. Please ensure that is defined the first time it is used.

Senior Editor:

Thank you for submitting your manuscript for consideration by The Journal of Physiology. As part of the peer review process, we recruited two Referees with expertise in this field of study. As a result, you now have two detailed sets of critiques along with a summary from our Reviewing Editor. Both Referees indicated that the manuscript has the potential to be influential in this field of study, but only if a number of major concerns are addressed. At this point we would like to invite you to respond point-by-point to each Referee's concerns, making corresponding revisions to your manuscript. Please also bring the revised paper into compliance with The Journal's statistics policy. We look forward to seeing your revised work and appreciate your interest in The Journal of Physiology.

Please revisit The Journal statistical reporting policy to ensure compliance. In particular, please include precise p-values in the figures. Thank you!

REFEREE COMMENTS

Referee #1:

General assessment

This manuscript addresses a timely and practically relevant question: whether combining sprint interval training (SIT) with post-exercise blood flow restriction (BFR) modifies skeletal muscle mitochondrial markers and endurance-related performance outcomes. The experimental approach-integrating pre/post performance testing with muscle biopsies and high-resolution respirometry-is ambitious and, in principle, well-suited to interrogate potential mechanistic adaptations. The

Methods are generally detailed, and the study has clear translational relevance for practitioners considering BFR as an adjunct to interval training.

Several points would benefit from clarification to improve internal consistency of the rationale, transparency of allocation procedures, and reproducibility of key methodological steps. In addition, a few statements in the Abstract/Discussion overstate conclusions relative to the statistical results and should be tempered.

Specific comments (by line)

Line 53-55: The phrasing is currently grammatically incomplete ("not tolerable ...") and "these responses" is ambiguous (BFR-induced vs. higher-intensity-induced responses). Consider revising along the following lines: "Blood flow restriction (BFR) during exercise can augment physiological adaptations and improve performance. However, the higher intensities typically required to elicit comparable adaptations are often not tolerable when performed concurrently with BFR."

Line 55-58: The rationale should be clarified. Stating that post-exercise BFR "does not limit training intensity" implies that preserving high intensity is the key advantage, which is not fully consistent with the established premise of BFR (i.e., eliciting adaptations at lower external loads/intensities). Please clarify the specific added value of post-exercise BFR when combined with SIT (i.e., augmentation beyond SIT at the same SIT dose), rather than framing it primarily as maintaining training intensity.

Line 88-89: The claim that "intensity appears to be more important" for improvements in mitochondrial respiration is not intuitive and may read contradictory: from a classical mitochondrial-adaptation perspective, one would rather expect low-intensity, high-volume work (high cumulative oxidative flux/time-at-task) to be the more "logical" driver. Please justify this statement explicitly by adding a short mechanistic rationale (and clarify what you mean by "intensity" vs "volume," and the intensity domain), or soften/rephrase the conclusion to reflect the nuance in the cited literature.

Line 102-103: Please consider adding that BFR has also been applied in sprint-based protocols, with reports of performance improvements, to complement the example from low-intensity running. For instance, include the sprint BFR study reporting improved performance (PMID: 27941491).

Line 133 and Line 161: The cycling modality should be made explicit much earlier-ideally in the title and/or Introduction. At present, readers only learn in the Methods that the SIT and testing were performed on a cycle ergometer, which delays proper interpretation of the study context and transferability. Please specify upfront that this is cycle-ergometer SIT (and, if applicable, that outcomes are cycling-specific).

Line 135-136: The wording "muscle biopsy screening questionnaires" is unclear. Please specify what this screening entailed and distinguish it from the general health screening used to identify contraindications to maximal exercise and BFR.

Line 145-149: Although allocation was based on baseline $\dot{V}O_2$peak and maximal aerobic power (MAP), the exact procedure remains unclear. Please specify whether participants were pair-/strata-matched on these variables and then randomized within pairs/strata, or whether assignment was deterministic or investigator-decided. Also clarify who performed the allocation and whether any allocation concealment was used.

Line 161-163: Please provide more detail on the cycle ergometer setup/standardization. You state that handlebar and saddle dimensions were recorded and kept constant, but it is unclear how these were set initially and which parameters were recorded (e.g., saddle height, saddle fore-aft, handlebar height/reach).

Line 166: Please clarify whether other supplements and ergogenic aids were controlled. You instruct participants to avoid alcohol and caffeine for 48 h, but it is not stated whether they were also asked to refrain from dietary supplements/ergogenic aids and/or whether habitual use was recorded and standardized.

Line 173: The description of sprint resistance is ambiguous and appears dimensionally inconsistent ("0.7 × body mass = braking torque"). Please specify units and clarify whether this refers to a manufacturer-specific torque-factor setting on the cycle ergometer versus an externally derived torque.

Line 205: Due to technical issues only 12 participants completed the lactate threshold test as described above. Please specify the nature of the technical issues, indicate whether failures occurred pre- or post-intervention, and report how many participants were affected in each group.

Line 210: Please clarify whether participants' habitual training was monitored and recorded during the intervention period. Without monitoring, adherence to the "no HIIT" instruction and potential between-group differences in background training remain uncertain.

Line 230-236: Please provide more methodological detail on the biopsy instrumentation. You describe a percutaneous needle biopsy with suction, but the needle type and specifications are not reported. Please state the biopsy needle used (e.g., Bergström or microbiopsy needle), including gauge/diameter (and manufacturer/model if possible).

Line 258: Briefly clarify how O_2 setpoints were controlled/calibrated (air calibration, barometric pressure, background correction).

Line 272: Please report how many samples exceeded this threshold and whether any were excluded.

Line 275: FCCP dose seems incorrect: "0.75-1.5 mM" is very likely a typo (typically µM). Please check.

Line 332: The term "CS activity-corrected" is not defined. Please clarify exactly how citrate synthase (CS) activity was used to correct mitochondrial respiration.

Line 351-352: The "main findings" statement is overstated. While the significant time × condition interaction supports a different pattern of change between groups, describing "no change with SIT alone" is not justified given the borderline P value and large ES in CON. Please rephrase to focus on the interaction (change differed by condition), avoid implying an overall time/condition effect (main effects were non-significant), and soften "reflecting increased mitochondrial content" as CS is an indirect proxy.

Referee #2:

Sprint Interval Training with Post Exercise Blood Flow Restriction Increases Mitochondrial Content and Respiration

This study examined whether adding post exercise blood flow restriction to a six-week sprint interval training program augments mitochondrial adaptations beyond sprint interval training alone in physically active men. The primary outcomes were citrate synthase activity as a marker of mitochondrial content and high resolution respirometry based indices of mitochondrial respiratory capacity. The authors report that BFR increased citrate synthase activity and augmented selected

respiratory variables, while changes in VO2peak and lactate threshold measures were not clearly different between groups.

Overall, I view this as an important and well conceived study that builds logically on prior work from this group. Assessing both mitochondrial content and function is a clear strength, and the physiological methods used are appropriate and rigorous. The training intervention is well-controlled and the mechanistic focus is valuable. However, several issues related to statistical interpretation and outcome selection limit the influence and impact of the paper in its current form. These concerns should be addressed to ensure the conclusions are aligned with the strength of the evidence.

General Comments

The unequal group sizes (BFR n = 12, CON n = 8) represent a meaningful limitation, and it is unclear to this reviewer why the groups were not balanced during allocation. While the repeated measures ANOVA is appropriate, unequal allocation reduces power and precision for detecting time by condition interactions and increases the likelihood of asymmetric inference. In practical terms, estimates in the larger BFR group are more stable, making significant within group changes more likely to emerge there than in the smaller control group.

In this context, conclusions framed as "BFR increased while CON did not" must be anchored primarily to a statistically supported interaction effect. Without a robust interaction, reliance on within group post-hoc tests risks overstating the differential effect of BFR.

Relatedly, the tendency to present post-hoc within group changes in a way that reads as evidence of between group differences, even when the ANOVA does not support a significant interaction, skews interpretation and creates the appearance of conflicting results. This is particularly problematic in the setting of unbalanced groups, where divergence in statistical significance may simply reflect differences in variance and sample size rather than a true physiological effect. In practical terms, the unequal allocation skews power toward being able to detect a significant within group effect in BFR over CON given the same absolute change and variability, because the larger sample provides more stable estimates. The Results narrative around line 346 illustrates this concern: the wording implies that certain physiological variables increased in BFR but not in CON, despite the earlier statement that there were no time by condition interactions for these measures. This framing gives the impression of a differential effect that is not supported by the primary inferential test. I would recommend revising this section so that claims of augmentation by BFR are limited to outcomes with a supported interaction, and that other changes are described as overall time effects without implying group superiority.

When an interaction is present, interpretation should focus on that interaction. It is not necessary to emphasize main effects of time for the same outcome, and doing so can dilute clarity.

The study is framed around endurance relevant mitochondrial adaptations, yet the performance outcomes are limited. The only direct performance metrics are sprint power variables, which are unlikely to be sensitive to changes in aerobic capacity in this context. While VO2peak and lactate threshold measures are useful physiological markers and may relate to performance, they are not direct performance outcomes. The absence of an aerobically specific test, such as a time trial or constant load tolerance assessment, limits the translational impact of the findings. This does not detract from the mechanistic contribution of the work, but it should temper statements regarding practical performance implications, particularly in the absence of clear between group differences in these physiological measures.

The choice to report VO2peak rather than VO2max is somewhat unusual in a study drawing physiological conclusions about adaptation, in young trained cyclists. A verified VO2max, supported by established secondary criteria, would provide stronger objective evidence of maximal oxygen transport capacity. Have the authors examined whether participants met accepted maximal criteria at pre and post testing, and how many did so? Given the variability in responses and the reductions observed in some individuals, without confirmation of maximal attainment these decreases cannot confidently be interpreted as physiological regressions rather than reflecting motivation, pacing, or residual fatigue.

Specific Comments and Minor Edits

Abstract: Lines 35 and 49 are awkwardly phrased. The wording could be simplified for clarity, for example by using "a six week SIT program with or without post exercise BFR."

Key Points: The second sentence is awkwardly constructed and difficult to follow. It would benefit from restructuring for clarity.

Introduction: Line 117 contains awkward phrasing in the sentence beginning "despite an increase..." and should be tightened.

Results: Line 346 should be revised to avoid implying a between group difference on the basis of post hoc within group changes when no interaction was detected. This is an important correction.

Discussion: Line 442 contains a misspelling of "Mitchell."

END OF COMMENTS

EDITOR COMMENTS

Reviewing Editor:
Thank you for your submission. Both referees highlighted the importance of your investigation and the methodological approach employed. Both referees had salient suggestions in improving the manuscript and highlighted a couple weaknesses. Please address all of them.

We thank the editors and reviewers for handling and reviewing this manuscript, appreciating the importance of the work, and the points raised to improve the clarity of the conclusions from these data.

Please pay particular attention the valid concerns raised regarding the imbalance sample sizes, post-hoc analyses, and interpretation of the interaction between time and training modality. If the authors believe that the imbalance between intervention groups does not influence the results, they should provide a robust counter-argument to these concerns.

We accept the unbalanced groups in this study is a limitation which we have directly addressed in both the responses to reviewer comments and the revised manuscript.

This imbalance is a result of an initial, wider study, which included an additional experimental group incorporating a deconditioning phase following a 6-week SIT+BFR intervention. Due to feasibility challenges, the deconditioning phase of the study was discontinued. However, 4 participants had completed the 6-week SIT+BFR intervention, which was identical to the present study. These data were subsequently included in the BFR group of the present study, resulting in the additional 4 participants compared with CON. Overall, whilst this imbalance is a limitation, we do not believe this influences the key results or conclusions of the research. However, we do agree that the manuscript requires clearer and tempered terminology to indicate the use of statistics and post-hoc analyses in comparisons made within groups, based on time effects. We also agree that this terminology should be more overtly distinct from that used to describe conclusions based on post-hoc analyses used to further investigate interaction effects, for transparency and clarity. We are grateful to the reviewers for highlighting this and accordingly, have amended these statements throughout.

The authors use CS activity as a means of estimating mitochondrial content. While it is appreciated that in the past it has been used a valid marker of mitochondrial content in a limited cohort of individuals (PMID: 22586215), more recent investigations raise cause for concern (r=0460, PMID: 32255679) and (r=0.546, PMID: 28580772). Additionally, a change in CS activity fails to correlate/predict changes in mitochondrial volume (PMID: 28580772) particularly after exercise training. Given recent data, it seems inappropriate to use CS

activity as a marker of content and/or use it as a means to evaluate intrinsic mitochondrial capacity.

We thank the editor for highlighting this important consideration in the validity of CS activity as a marker of mitochondrial content, specifically. We accept that using an indirect biomarker of transmission electron microscopy to examine mitochondrial content indirectly is inherently associative. Accordingly, we have included the research cited by the editor, and some discussion of their findings, to present a holistic examination of what CS activity represents, including difficulties distinguishing between mitochondrial enlargement and functional mass (matrix volume), compared with direct assessment of mitochondrial content, specifically.

Whilst there are small sample sizes in the cited literature examining correlations between CS activity and mitochondrial content, the cohorts in that literature are active, young healthy males, comparable with the cohort in the present study (moderately trained, healthy males). Importantly, the conclusion of the authors in the study cited by the editor (PMID: 28580772), where no significant correlation between changes in CS activity and mitochondrial content were found with training indicate that CS activity is a valid marker for changes in mitochondrial volume density, if not content, *per se*. This is likely due to the correlations evident at baseline and following the training intervention. A detailed discussion of this in the context of our data is provided in the revised manuscript (Lines 393 - 404) and we believe this transparently clarifies the findings of the present study.

Minor comments/ considerations:

The authors should also consider to expand their analysis of their respirometry data by evaluating flux control ratios (P/E, L/E, L/P).

We thank the editor for this comment. This analysis was conducted and we observed there was a condition x time interactions ($P = 0.036$) for the inverse respiratory control ratio, but no differences were found with post-hoc t-tests in either BFR or CON groups ($P \geq 0.068$). No further time, group or interaction effects were observed.

We believe the main effect of time in both coupled and uncoupled respiration in the present study indicates a comparable increase in mitochondrial respiration across several parameters, resulting in the lack of changes in these ratios following training, despite the absolute changes. Furthermore, a lack of change in flux control ratios concomitant with enhanced electron transfer system capacity has previously been reported in the response to training (PMID: 28580772).

Whilst we initially felt reluctant to report additional mitochondrial normalisations of the same data without cause, and that this data was not novel, on consideration, the lack of change in FCR with changes in CS activity in the present study, adds further depth. This may

allow for better interpretation of our observed effects, whereby the interpretation of this data could support morphological adaptations, in the present study. Therefore, we have included this data in the methods (Line 292 – 300) results (Line 355 – 360), added a table (Tabe 3), and incorporated these data into the discussion of the mitochondrial biogenesis observed in the present study (Line 444 – 460).

The authors presumably use "ES" as an abbreviation for "Effect Size" in the results. Please ensure that is defined the first time it is used.

Added at first mention (Line 321).

Senior Editor:

Thank you for submitting your manuscript for consideration by The Journal of Physiology. As part of the peer review process, we recruited two Referees with expertise in this field of study. As a result, you now have two detailed sets of critiques along with a summary from our Reviewing Editor. Both Referees indicated that the manuscript has the potential to be influential in this field of study, but only if a number of major concerns are addressed. At this point we would like to invite you to respond point-by-point to each Referee's concerns, making corresponding revisions to your manuscript. Please also bring the revised paper into compliance with The Journal's statistics policy. We look forward to seeing your revised work and appreciate your interest in The Journal of Physiology.

Please revisit The Journal statistical reporting policy to ensure compliance. In particular, please include precise p-values in the figures. Thank you!

We thank the senior editor for their handling of this manuscript and for their invitation to address these comments and resubmit. All figures have also been adjust to include specific *P*-values, in compliance with the Journal of Physiology's statistics policy.

REFEREE COMMENTS

Referee #1:

General assessment

This manuscript addresses a timely and practically relevant question: whether combining sprint interval training (SIT) with post-exercise blood flow restriction (BFR) modifies skeletal muscle mitochondrial markers and endurance-related performance outcomes. The

experimental approach-integrating pre-/post-performance testing with muscle biopsies and high-resolution respirometry is ambitious and, in principle, well-suited to interrogate potential mechanistic adaptations. The Methods are generally detailed, and the study has clear translational relevance for practitioners considering BFR as an adjunct to interval training.

We thank the reviewer for the recognition of the methodological robustness of this study and the relevance of the findings in the context of the existing literature.

Several points would benefit from clarification to improve internal consistency of the rationale, transparency of allocation procedures, and reproducibility of key methodological steps. In addition, a few statements in the Abstract/Discussion overstate conclusions relative to the statistical results and should be tempered.

We thank the reviewer for highlighting the need for consistency of the rationale and procedures for the reader. We have addressed the specific comments to clarify the methodology and temper the conclusions, and we agree this improves the clarity of the manuscript.

Specific comments (by line)

Line 53-55: The phrasing is currently grammatically incomplete ("not tolerable ...") and "these responses" is ambiguous (BFR-induced vs. higher-intensity-induced responses). Consider revising along the following lines: "Blood flow restriction (BFR) during exercise can augment physiological adaptations and improve performance. However, the higher intensities typically required to elicit comparable adaptations are often not tolerable when performed concurrently with BFR."

We thank the reviewer for this comment. On reflection, we feel that this statement does not reflect the key points of this study, rather the previous literature and have opted to omit this. The topic is still discussed in the introduction, but the intensity aspect of exercise with BFR used during compared with post-exercise BFR in sprint interval training is not specifically addressed in the experimental design of this study.

Line 55-58: The rationale should be clarified. Stating that post-exercise BFR "does not limit training intensity" implies that preserving high intensity is the key advantage, which is not fully consistent with the established premise of BFR (i.e., eliciting adaptations at lower external loads/intensities). Please clarify the specific added value of post-exercise BFR when combined with SIT (i.e., augmentation beyond SIT at the same SIT dose), rather than framing it primarily as maintaining training intensity.

We agree that the augmentation of the exercise stimulus beyond that in work-matched training is the essential premise of BFR. However, we maintain that the advantage and

benefit of the post-exercise BFR method utilised in the present study, in comparison with the literature examining low-intensity exercise with BFR, is that it allows for intensity-dependent adaptation to be maintained, whilst allowing for extended exposure to exercise-induced homeostatic perturbations. This has been established in previous literature examining SIT with post-exercise BFR, cited in the introduction and is also observed in the results of the present study. However, we have amended the statement to clarify this rationale in line with this comment (Line 56 – 67). We have also removed the previous statement to avoid over focussing on the intensity debate in the key points, as highlighted in the previous comment.

Line 88-89: The claim that "intensity appears to be more important" for improvements in mitochondrial respiration is not intuitive and may read contradictory: from a classical mitochondrial-adaptation perspective, one would rather expect low-intensity, high-volume work (high cumulative oxidative flux/time-at-task) to be the more "logical" driver. Please justify this statement explicitly by adding a short mechanistic rationale (and clarify what you mean by "intensity" vs "volume," and the intensity domain), or soften/rephrase the conclusion to reflect the nuance in the cited literature.

We agree that the debate surrounding the role of intensity as a driver for improvements in mitochondrial respiration, specifically, as opposed to the classical mitochondrial-adaptation perspective for mitochondrial biogenesis (primarily linked to changes in mitochondrial content) is nuanced. Therefore, we have expanded this statement and included additional research to briefly indicate the mechanistic rationale (Line 88 – 91).

We have also taken the opportunity to expand on this with the discussion of recently published work (Line 469 – 474 and 481 – 483).

Line 102-103: Please consider adding that BFR has also been applied in sprint-based protocols, with reports of performance improvements, to complement the example from low-intensity running. For instance, include the sprint BFR study reporting improved performance (PMID: 27941491).

We thank the reviewer for their comment and for highlighting this work. This study used ‘low-intensity sprint exercise’, in a work-matched fashion, with the BFR applied during the sprints, which presumably limited the workload achievable during BFR (hence the 60 – 70% maximal velocity used in both groups). We question whether submaximal training is truly ‘sprint’ exercise. Nevertheless, given the higher intensity of this repeated sprint-style protocol, and the apparent relevance of improved sprint performance, in both groups, to the present study, we have added to the discussion of performance parameters later in the manuscript (Line 499).

Line 133 and Line 161: The cycling modality should be made explicit much earlier-ideally in the title and/or Introduction. At present, readers only learn in the Methods that the SIT and

testing were performed on a cycle ergometer, which delays proper interpretation of the study context and transferability. Please specify upfront that this is cycle-ergometer SIT (and, if applicable, that outcomes are cycling-specific).

We thank the reviewer for this comment and have included the cycling format of the training and performance assessments in the introduction (Line 126 and 132).

Line 135-136: The wording "muscle biopsy screening questionnaires" is unclear. Please specify what this screening entailed and distinguish it from the general health screening used to identify contraindications to maximal exercise and BFR.

We thank the reviewer for this comment. These are questions within the standard health screen questionnaire. We have amended the statement to reflect that this screening was not separate, but latterly, to mitigate contraindications to administration of lidocaine hydrochloride local anaesthetic (Line 139 – 140).

Line 145-149: Although allocation was based on baseline $\dot{V}O_2$peak and maximal aerobic power (MAP), the exact procedure remains unclear. Please specify whether participants were pair-/strata-matched on these variables and then randomized within pairs/strata, or whether assignment was deterministic or investigator-decided. Also clarify who performed the allocation and whether any allocation concealment was used.

We have clarified the allocation procedures as indicated (Line 152).

Line 161-163: Please provide more detail on the cycle ergometer setup/standardization. You state that handlebar and saddle dimensions were recorded and kept constant, but it is unclear how these were set initially and which parameters were recorded (e.g., saddle height, saddle fore-aft, handlebar height/reach).

We thank the reviewer for this comment and have specified the dimensions and their self-selected nature (Line 165 – 166).

Line 166: Please clarify whether other supplements and ergogenic aids were controlled. You instruct participants to avoid alcohol and caffeine for 48 h, but it is not stated whether they were also asked to refrain from dietary supplements/ergogenic aids and/or whether habitual use was recorded and standardized.

We did not ask participants to record or standardise their use of dietary supplements and given the breadth of potentially ergogenic supplements, we did not record this. We only asked that participants maintain their normal diet, inclusive of any food-based supplements, which we have specified directly (Line 169).

Line 173: The description of sprint resistance is ambiguous and appears dimensionally

inconsistent ("0.7 × body mass = braking torque"). Please specify units and clarify whether this refers to a manufacturer-specific torque-factor setting on the cycle ergometer versus an externally derived torque.

We thank the reviewer for this suggestion which we have adopted to remove ambiguity (Line 178 – 179).

Line 205: Due to technical issues only 12 participants completed the lactate threshold test as described above. Please specify the nature of the technical issues, indicate whether failures occurred pre- or post-intervention, and report how many participants were affected in each group.

These technical issues occurred pre-intervention due to issues with the equipment, and we have amended this statement to clarify this (Line 208). Participant *n* for this assessment is already provided for both groups, which sufficiently indicates the number of participants affected (208 – 209).

Line 210: Please clarify whether participants' habitual training was monitored and recorded during the intervention period. Without monitoring, adherence to the "no HIIT" instruction and potential between-group differences in background training remain uncertain.

We thank the reviewer for this comment. Habitual activity was not monitored. Adherence to study standardisations, including training and diet are inherently uncertain in any research without monitoring, however, this was not feasible in this study. Participants were performing three sessions per week of sprint intervals in both intervention groups on the study. In any training block in a moderately trained cohort, this would represent a significant frequency of high-intensity exercise, sufficient or beyond that of what one may typically expect. Therefore, whilst it remains uncertain, the regular contact and conversation with participants during supervised training involved frequent discussion of their training outside of the laboratory, allowing us to be as confident as is possible by self-report, that these requirements were adhered to.

Line 230-236: Please provide more methodological detail on the biopsy instrumentation. You describe a percutaneous needle biopsy with suction, but the needle type and specifications are not reported. Please state the biopsy needle used (e.g., Bergström or microbiopsy needle), including gauge/diameter (and manufacturer/model if possible).

We have provided specific detail of the biopsy needle instrument (Line 236).

Line 258: Briefly clarify how O_2 setpoints were controlled/calibrated (air calibration, barometric pressure, background correction).

We have included these details of calibration which reflect manufacturer instruction for clarity (Line 264 – 267).

Line 272: Please report how many samples exceeded this threshold and whether any were excluded.

We have included this detail as requested (Line 279).

Line 275: FCCP dose seems incorrect: "0.75-1.5 mM" is very likely a typo (typically µM). Please check.

We thank the reviewer for correctly noting this typo, which we have amended (Line 282).

Line 332: The term "CS activity-corrected" is not defined. Please clarify exactly how citrate synthase (CS) activity was used to correct mitochondrial respiration.

We have clarified that this statement means expressed relative to CS activity and defined here for subsequent use (Line 345 – 346).

Line 351-352: The "main findings" statement is overstated. While the significant time × condition interaction supports a different pattern of change between groups, describing "no change with SIT alone" is not justified given the borderline P value and large ES in CON. Please rephrase to focus on the interaction (change differed by condition), avoid implying an overall time/condition effect (main effects were non-significant), and soften "reflecting increased mitochondrial content" as CS is an indirect proxy.

We thank the reviewer for these comments. The has a borderline *P*-value and large effect size in CON is indicative of a trend towards a decrease in CS activity, compared with a significant increase in BFR. Therefore, we are confident in the conclusion that there is an increase in BFR, compared with CON. We also feel 'no change', rather than implying a decrease due to the tendency and the large effect size in CON better reflects our data, given the variance of inter-biopsy analysis of CS activity (PMID: 34985815). Furthermore, the Journal Statistics policy prohibits the discussion of trends. However, in the interest of clarity, we have removed 'no change' from the main findings statement, in favour of 'compared with SIT alone, to ensure the data is not represented.

Considering the time*condition effect, the post-hoc analysis of the interaction effect stated demonstrates an increase in BFR (P = 0.040; ES = 0.68). It is unsurprising there is no main effect of time when these data are grouped, given the differential pattern of response between conditions. Therefore, we feel that the importance of this finding in the context of the potential for BFR to induce increases in CS activity compared with SIT alone is not overstated. The lack of increase in response to SIT in CON is also discussed at length in the discussion (Line 404 – 412).

We have softened the language related to CS activity in the main findings (Line 371) as well as adding a detailed assessment of the validity of this marker in the discussion (Line 393 – 404).

Referee #2:

Sprint Interval Training with Post Exercise Blood Flow Restriction Increases Mitochondrial Content and Respiration

This study examined whether adding post exercise blood flow restriction to a six-week sprint interval training program augments mitochondrial adaptations beyond sprint interval training alone in physically active men. The primary outcomes were citrate synthase activity as a marker of mitochondrial content and high resolution respirometry based indices of mitochondrial respiratory capacity. The authors report that BFR increased citrate synthase activity and augmented selected respiratory variables, while changes in VO2peak and lactate threshold measures were not clearly different between groups.

Overall, I view this as an important and well conceived study that builds logically on prior work from this group. Assessing both mitochondrial content and function is a clear strength, and the physiological methods used are appropriate and rigorous. The training intervention is well-controlled and the mechanistic focus is valuable. However, several issues related to statistical interpretation and outcome selection limit the influence and impact of the paper in its current form. These concerns should be addressed to ensure the conclusions are aligned with the strength of the evidence.

We thank the reviewer for these comments and the recognition of the importance and methodological rigour of this study. We have addressed the comments regarding statistics and clarified the interpretation of the findings to ensure that the conclusions are aligned with the statistical assessments of the data.

General Comments

The unequal group sizes (BFR n = 12, CON n = 8) represent a meaningful limitation, and it is unclear to this reviewer why the groups were not balanced during allocation. While the repeated measures ANOVA is appropriate, unequal allocation reduces power and precision for detecting time by condition interactions and increases the likelihood of asymmetric inference. In practical terms, estimates in the larger BFR group are more stable, making significant within group changes more likely to emerge there than in the smaller control group.

In this context, conclusions framed as "BFR increased while CON did not" must be anchored

primarily to a statistically supported interaction effect. Without a robust interaction, reliance on within group post-hoc tests risks overstating the differential effect of BFR.

We accept the unbalanced groups in this study is a limitation which we have directly addressed in both the responses to reviewer comments and the revised manuscript.

This imbalance is a result of an initial, wider study, which included an additional experimental group incorporating a deconditioning phase following a 6-week SIT+BFR intervention. Due to feasibility challenges, the deconditioning phase of the study was discontinued. However, 4 participants had completed the 6-week SIT+BFR intervention, which was identical to the present study. These data were subsequently included in the BFR group of the present study, resulting in the additional 4 participants compared with CON. Overall, whilst this imbalance is a limitation, we do not believe this influences the key results or conclusions of the research. However, we do agree that the manuscript requires clearer and tempered terminology to indicate the use of statistics and post-hoc analyses in comparisons made within groups, based on time effects. We also agree that this terminology should be more overtly distinct from that used to describe conclusions based on post-hoc analyses used to further investigate interaction effects, for transparency and clarity. We are grateful to the reviewers for highlighting this and accordingly, have amended these statements throughout.

We do not believe that the imbalance effects the conclusions of this study. However, we recognise the additional numbers in the BFR group may bias the likelihood of finding a significant effect of time, and accordingly, have clarified and focussed discussion and the conclusions on interaction effects, and where time effects are explored, we been clear in highlighting this and have tempered the language to avoid overstating any conclusions arising from this statistical interpretation.

Relatedly, the tendency to present post-hoc within group changes in a way that reads as evidence of between group differences, even when the ANOVA does not support a significant interaction, skews interpretation and creates the appearance of conflicting results. This is particularly problematic in the setting of unbalanced groups, where divergence in statistical significance may simply reflect differences in variance and sample size rather than a true physiological effect. In practical terms, the unequal allocation skews power toward being able to detect a significant within group effect in BFR over CON given the same absolute change and variability, because the larger sample provides more stable estimates. The Results narrative around line 346 illustrates this concern: the wording implies that certain physiological variables increased in BFR but not in CON, despite the earlier statement that there were no time by condition interactions for these measures. This framing gives the impression of a differential effect that is not supported by the primary inferential test. I would recommend revising this section so that claims of augmentation by BFR are limited to outcomes with a supported interaction, and that other changes are

described as overall time effects without implying group superiority.

When an interaction is present, interpretation should focus on that interaction. It is not necessary to emphasize main effects of time for the same outcome, and doing so can dilute clarity.

We thank the reviewer for this comment and have similarly tempered the prose used in the discussion of within group comparisons to avoid the implications of differential effects between groups, except in cases where interaction effects are present (Line 419 – 420 and Line 427). In those cases where interaction effects are present, the focus in the discussion has been altered to discuss these specifically, and we agree these alterations improve the clarity of the conclusions. However, the line cited (Line 371) refers to CS activity, where an interaction effect and post-hoc t-test demonstrate differences between conditions over time.

We have also highlighted in the discussion and the methods specifically that the respirometry analysis was conducted in smaller, but more comparable sample sizes, where examinations of the post-hoc t-tests, sample sizes and main effects may be more relevant (Line 433 – 434). However, we have maintained clarity of which effects are being discussed and tempered language relating to time effects.

The study is framed around endurance relevant mitochondrial adaptations, yet the performance outcomes are limited. The only direct performance metrics are sprint power variables, which are unlikely to be sensitive to changes in aerobic capacity in this context. While VO2peak and lactate threshold measures are useful physiological markers and may relate to performance, they are not direct performance outcomes. The absence of an aerobically specific test, such as a time trial or constant load tolerance assessment, limits the translational impact of the findings. This does not detract from the mechanistic contribution of the work, but it should temper statements regarding practical performance implications, particularly in the absence of clear between group differences in these physiological measures.

We thank the reviewer for these comments and recognise that the exercise performance outcomes/physiological markers would benefit from the addition of a performance specific endurance test. The primary aim of this study was to examine the mechanistic basis of previously reported improvements in $\dot{V}O_2$peak, and we appreciate the recognition of this contribution; accordingly, we have tempered the practical implications to focus on the changes in physiological markers, rather than performance *per se*, opting for the terminology of ‘markers of (endurance) performance’ for transparency (Line 501 – 502, 504, 518 and 525). Additionally, we have removed comparisons between groups for time effects, to discuss an overall improvement. (Line 506)

The choice to report VO2peak rather than VO2max is somewhat unusual in a study drawing

physiological conclusions about adaptation, in young trained cyclists. A verified VO2max, supported by established secondary criteria, would provide stronger objective evidence of maximal oxygen transport capacity. Have the authors examined whether participants met accepted maximal criteria at pre and post testing, and how many did so? Given the variability in responses and the reductions observed in some individuals, without confirmation of maximal attainment these decreases cannot confidently be interpreted as physiological regressions rather than reflecting motivation, pacing, or residual fatigue.

The nature of the RAMP test employed means that the assessment of a traditional plateau as the primary criteria for VO_2max, without a verification phase, was not possible. Additionally, given that a sprint test was performed on the same day during the same session as the graded-exercise test to exhaustion, the addition of a verification phase was deemed unfeasible, given the already high demands on the participant. We accept this is a limitation of the findings.

Whilst secondary criteria were met by participants pre- and post-training to satisfy the requirement for the attainment of VO_2peak, we are reluctant to define this as VO_2max without the verification test, to avoid overstating our data. We have included this consideration in the manuscript fort clarity (Line 508 – 512).

Specific Comments and Minor Edits

Abstract: Lines 35 and 49 are awkwardly phrased. The wording could be simplified for clarity, for example by using "a six week SIT program with or without post exercise BFR."

We thank the reviewer for this suggestion which we have utilised. (Line 35 and 49)

Key Points: The second sentence is awkwardly constructed and difficult to follow. It would benefit from restructuring for clarity.

We have amended this sentence for clarity. (Line 53 – 55)

Introduction: Line 117 contains awkward phrasing in the sentence beginning "despite an increase..." and should be tightened.

We thank the reviewer for this comment and agree that the structure of this sentence was a little awkward, so have rephrased. (Line 119 -120)

Results: Line 346 should be revised to avoid implying a between group difference on the basis of post hoc within group changes when no interaction was detected. This is an important correction.

Line 346 (now Line 370 – 371) refers to the increased CS activity, compared with no change in CON, where a significant interaction effect was detected. The discussion has been checked throughout to ensure clarity of conclusions relating to interactions and within measures statistics.

Discussion: Line 442 contains a misspelling of "Mitchell."

We thank the reviewer for highlighting this typo, which has been amended (Line 500).

Dear Dr Peden,

Re: JP-RP-2026-290955R1 "**Sprint-Interval Training with Post-Exercise Blood Flow Restriction Increases Mitochondrial Content and Respiration**" by Donald Lang Peden, Carolin Stangier, Emma Mitchell, Stephen J Bailey, and Richard A. Ferguson

Thank you for submitting your manuscript to The Journal of Physiology. It has been assessed by a Reviewing Editor and by 2 expert referees and we are pleased to tell you that it is acceptable for publication following satisfactory revision.

The referee reports are copied at the end of this email.

Please address all the points raised and incorporate all requested revisions or explain in your Response to Referees why a change has not been made. We hope you will find the comments helpful and that you will be able to return your revised manuscript within 4 weeks. If you require longer than this, please contact journal staff: jp@physoc.org.

Your revised manuscript should be submitted online using the link in your Author Tasks Link Not Available. This link is accessible via your account as Corresponding Author; it is not available to your co-authors. If this presents a problem, please contact journal staff (jp@physoc.org). Image files from the previous version are retained on the system. Please ensure you replace or remove any files that are being revised.

If you do not wish to submit a revised version of your manuscript, you must inform our journal staff (jp@physoc.org) or reply to this email to request withdrawal. Please note that a manuscript must be formally withdrawn from the peer review process at one journal before it may be submitted to another journal.

TRANSPARENT PEER REVIEW POLICY: To improve the transparency of its peer review process The Journal of Physiology publishes online, as supporting information, the peer review history of all articles accepted for publication. Readers will have access to decision letters, including Editors' comments and referee reports, for each version of the manuscript, as well as any author responses to peer review comments. Referees can decide whether or not they wish to be named on the peer review history document.

ABSTRACT FIGURES: Authors are expected to use The Journal's premium BioRender account to create/redraw their Abstract Figures. Information on how to access this account is here: https://physoc.onlinelibrary.wiley.com/journal/14697793/biorender-access.

This will enable Authors to create and download high-resolution figures. If authors have used the free BioRender service, they can use the instructions provided in the link above to download a high-resolution version suitable for publication.

The link provided should only be used for the purposes of this submission. Authors will be charged for figures created on this account if they are not related to this manuscript submission.

LANGUAGE EDITING AND SUPPORT FOR PUBLICATION: If you would like help with English language editing, or other article preparation support, Wiley Editing Services offers expert help, including English Language Editing, as well as translation, manuscript formatting, and figure formatting at www.wileyauthors.com/eeo/preparation. You can also find resources for Preparing Your Article for general guidance about writing and preparing your manuscript at www.wileyauthors.com/eeo/prepresources.

RIGOUR AND REPRODUCIBILITY: We recommend authors consult The Journal's Rigour and Reproducibility requirements to ensure that their manuscript is suitable for publication: https://physoc.onlinelibrary.wiley.com/pb-assets/hub-assets/physoc/documents/TJP-Rigour-and-Reproducibility-Requirements.pdf

REVISION CHECKLIST:

Check that your Methods section conforms to journal policy: https://jp.msubmit.net/cgi-bin/main.plex?form_type=display_requirements#methods

Check that data presented conforms to the statistics policy: https://jp.msubmit.net/cgi-bin/main.plex?form_type=display_requirements#statistics

Upload a full Response to Referees file. To create your 'Response to Referees' copy all the reports, including any comments from the Senior and Reviewing Editors, into a Microsoft Word, or similar, file and respond to each point, using font or background colour to distinguish comments and responses and upload as the required file type.

Please upload two versions of your manuscript text: one with all relevant changes highlighted and one clean version with no changes tracked. The manuscript file should include all tables and figure legends, but each figure/graph should be uploaded as separate, high-resolution files. The journal is now integrated with Wiley's Image Checking service. For further details, see: https://www.wiley.com/en-us/network/publishing/research-publishing/trending-stories/upholding-image-integrity-wileys-

image-screening-service

You may also upload:

- 'Potential Cover Art' for consideration as the issue's cover image
- Appropriate Supporting Information (Video, audio or data set: see https://jp.msubmit.net/cgi-bin/main.plex?form_type=display_requirements#supp).

We look forward to receiving your revised submission.

If you have any queries, please reply to this email and we will be pleased to advise.

Yours sincerely,

Karyn Hamilton
Senior Editor
The Journal of Physiology

REQUIRED ITEMS

- Please include an Abstract Figure legend. An appropriate figure legend, which should not exceed 150 words in length, should be included in the main manuscript file.

- The corresponding author must provide an institutional email address (not a personal address) for their author account. We encourage ALL co-authors to also provide institutional email addresses. If this cannot be provided (as corresponding author), then a stamped letter must be provided from the institution which confirms their role and employment there (please upload this with the revised submission).

EDITOR COMMENTS

Reviewing Editor:

Thank you for your thoughtful and thorough revisions. Both reviewers found the revisions satisfactory and appreciated the acknowledgment of the imbalanced experimental groups as a limitation to the present study. There is an additional comment regarding the methodology/setting employed with the Lode Sport Excalibur.

Senior Editor:

Comments for Authors to ensure the paper complies with the Statistics Policy:
Thank you for providing precise p-values. Quick clarification. When you note, for example, "P {greater than or equal to} 0.135" do you really mean P = 0.135 ? In other words, why are you still noting {greater than or equal to} in some cases? Please revisit these incidences of statistical notation.

Comments to the Author:
Thank you for submitting your revised manuscript for continued consideration by The Journal of Physiology. The Referees were both complimentary about the improvements resulting from the revisions. Only a few minor suggestions and clarifications remain. At this point, we would like to provisionally accept the manuscript pending satisfactory revisions to address these few remaining points. We look forward to seeing your revisions soon. Thank you again!

REFEREE COMMENTS

Referee #1:

Overall, the revision is clearly improved and many of the requested clarifications have been implemented well (notably the

earlier specification of the cycling modality, enhanced methodological detail for biopsies and respirometry, and improved transparency around missing data). From my perspective, only one issue remains:

Sprint resistance (Lode Excalibur) reporting: The description of the Lode sprint resistance remains unclear and the units appear incorrect. "0.7 × body mass = braking torque ($\mathrm{N \cdot m^{-1}}$)" is dimensionally inconsistent; torque should be reported in N·m, and if a manufacturer-specific scaling was used it should be expressed as a torque factor (e.g., $\mathrm{N \cdot m \cdot kg^{-1}}$) with the corresponding formula (braking torque = torque factor × body mass). Please correct the units and report the exact Lode setting (mode/software parameter) to ensure reproducibility.

Referee #2:

The changes made by the authors have significantly improved the paper and are fair in recognizing the limitations. I have no further recommended changes.

END OF COMMENTS

EDITOR COMMENTS

Reviewing Editor:

Thank you for your thoughtful and thorough revisions. Both reviewers found the revisions satisfactory and appreciated the acknowledgment of the imbalanced experimental groups as a limitation to the present study. There is an additional comment regarding the methodology/setting employed with the Lode Sport Excalibur.

Thank you for the support throughout the review process and for assistance improving the manuscript. These final comments have been addressed.

Senior Editor:

Comments for Authors to ensure the paper complies with the Statistics Policy:
Thank you for providing precise p-values. Quick clarification. When you note, for example, "P {greater than or equal to} 0.135" do you really mean P = 0.135 ? In other words, why are you still noting {greater than or equal to} in some cases? Please revisit these incidences of statistical notation.

The adoption of ($P \geq 0.XXX$) has been used when multiple, non-significant statistical tests are referred to in text, and the smallest value is reported, to prevent repetition. The exact *P*-value for each test can be found in the cited table.

Comments to the Author:
Thank you for submitting your revised manuscript for continued consideration by The Journal of Physiology. The Referees were both complimentary about the improvements resulting from the revisions. Only a few minor suggestions and clarifications remain. At this point, we would like to provisionally accept the manuscript pending satisfactory revisions to address these few remaining points. We look forward to seeing your revisions soon. Thank you again!

We would like to thank both editors and reviewers for the timely and professional handling of this manuscript throughout the review process. We agree the manuscript has been improved by these revisions and have addressed the final suggestions and clarifications. We are pleased to have this work accepted for publication in this prestigious journal. Thank you again for your hard work.

REFEREE COMMENTS

Referee #1:

Overall, the revision is clearly improved and many of the requested clarifications have been implemented well (notably the earlier specification of the cycling modality, enhanced methodological detail for biopsies and respirometry, and improved transparency around missing data). From my perspective, only one issue remains:

Sprint resistance (Lode Excalibur) reporting: The description of the Lode sprint resistance remains unclear and the units appear incorrect. "0.7 × body mass = braking torque ($N \cdot m^{-1}$)" is dimensionally inconsistent; torque should be reported in N·m, and if a manufacturer-specific scaling was used it should be expressed as a torque factor (e.g., $N \cdot m \cdot kg^{-1}$) with the corresponding formula (braking torque = torque factor × body mass). Please correct the units and report the exact Lode setting (mode/software parameter) to ensure reproducibility.

We thank the reviewer for their comments regarding the revised manuscript. We recognise the need for further clarity for reproducibility and have removed any ambiguity in a revised description, with correct units (Line 171).

Referee #2:

The changes made by the authors have significantly improved the paper and are fair in recognizing the limitations. I have no further recommended changes.

We thank the reviewer for their comments regarding the revised manuscript.

Dear Dr Peden,

Re: JP-RP-2026-290955R2 "**Sprint-Interval Training with Post-Exercise Blood Flow Restriction Increases Mitochondrial Content and Respiration**" by Donald Lang Peden, Carolin Stangier, Emma Mitchell, Stephen J Bailey, and Richard A. Ferguson

We are pleased to tell you that your paper has been accepted for publication in The Journal of Physiology.

The last Word (or similar) version of the manuscript provided will be used by the Production Editor to prepare your proof. When this is ready you will receive an email containing a link to Wiley's Online Proofing System. The proof should be thoroughly checked and corrected as promptly as possible.

Authors should note that it is too late at this point to offer corrections prior to proofreading. Major corrections at proof stage, such as changes to figures, will be referred to the Editors for approval before they can be incorporated. Only minor changes, such as to style and consistency, should be made at proof stage. Changes that need to be made after proof stage will usually require a formal correction notice.

All queries at proof stage should be sent to: TJP@wiley.com.

Following copyediting and typesetting the accepted version will be published online.

Yours sincerely,

Karyn Hamilton
Senior Editor
The Journal of Physiology

IMPORTANT POINTS TO NOTE FOLLOWING ACCEPTANCE OF YOUR PAPER:

• IMPORTANT NOTICE ABOUT OPEN ACCESS: To assist authors whose funding agencies mandate immediate public access to published research findings, The Journal of Physiology allows authors to pay an Open Access (OA) fee to have their papers made freely available immediately on publication.

The Corresponding Author will receive an email from Wiley with details on how to register or log in to Wiley Authors where you will be able to place an order.

• You can check if your funder or institution has a Wiley Open Access Account here:
https://authors.wiley.com/author-resources/Journal-Authors/open-access/author-compliance-tool.html

• TRANSPARENT PEER REVIEW POLICY: To improve the transparency of its peer review process, The Journal of Physiology publishes online as supporting information the peer review history of all articles accepted for publication. Readers will have access to decision letters, including Editors' comments and referee reports, for each version of the manuscript as well as any author responses to peer review comments. Referees can decide whether or not they wish to be named on the peer review history document.

• You can help your research get the attention it deserves! Check out Wiley's free Promotion Guide for best-practice recommendations for promoting your work at: www.wileyauthors.com/eeo/guide. You can learn more about Wiley Editing Services which offers professional video, design, and writing services to create shareable video abstracts, infographics, conference posters, lay summaries, and research news stories for your research at: www.wileyauthors.com/eeo/promotion.

• If you would like to receive our 'Research Roundup', a monthly newsletter highlighting the cutting-edge research published in The Physiological Society's family of journals (The Journal of Physiology, Experimental Physiology, Physiological Reports, The Journal of Nutritional Physiology and The Journal of Precision Medicine: Health and Disease), please click this link, fill in your name and email address and select 'Research Roundup':
https://www.physoc.org/journals-and-media/membernews

EDITOR COMMENTS

Reviewing Editor:
Thank you for attending to the remaining comments.

Senior Editor:
Thank you for submitting your revised manuscript for continued consideration by The Journal of Physiology. We are pleased to accept it for publication in The Journal of Physiology. Thank you for your interest in The Journal and Congratulations!
